# Access to New Cytotoxic Quinone-Amino Acid Conjugates Linked through A Vinylic Spacer from 2-Acylnaphthoquinones and Methyl 3-Aminocrotonate

**DOI:** 10.3390/molecules22122281

**Published:** 2017-12-20

**Authors:** Jaime A. Valderrama, Joel Garrido, Virginia Delgado, Julio Benites, Cristina Theoduloz

**Affiliations:** 1 Instituto de Ciencias Exactas y Naturales (ICEN), Universidad Arturo Prat Casilla 121, Iquique 1100000, Chile; 2 Química y Farmacia, Facultad de Ciencias de la Salud, Universidad Arturo Prat, Casilla 121, Iquique 1100000, Chile; goejizz@gmail.com; 3 Facultad de Química, Pontificia Universidad Católica de Chile, Casilla 306, Santiago 6094411, Chile; vcdelgad@uc.cl; 4 Facultad de Ciencias de la Salud, Universidad de Talca, Talca 3460000, Chile; ctheodul@utalca.cl

**Keywords:** 2-acylnaphthoquinones, α-amino acid methyl esters, enaminones, quinone amino acid conjugates, cytotoxic activities

## Abstract

The reaction of 2-acetyl- and 2-benzoyl-1,4-naphthoquinone with (*Z*)-methyl 3-(hydroxymethyl)aminocrotonate proceeds through a formal [3+3] process to yield the corresponding 1,2-dihydrobenzisoquinolinequinones in 63% and 72% yield, respectively. The reactions of 2-acyl-1,4-naphthoquinone with enaminones, derived from diverse l- and d-amino acid methyl esters, produced the corresponding naphthoquinone amino acids conjugates bonded through a vinyl spacer in the yields range 40–71%. The presence of not-separable isomers of the naphthoquinone amino acids conjugates in the ^1^H- and ^13^C-NMR spectra is explained by the existence of conformational isomers generated by hindered rotation of the substituent bonded to the quinone double bond. These new naphthoquinone amino acids conjugates were screened in vitro on normal and cancer cell lines and showed moderate cytotoxic activities.

## 1. Introduction

**Figure 1:**
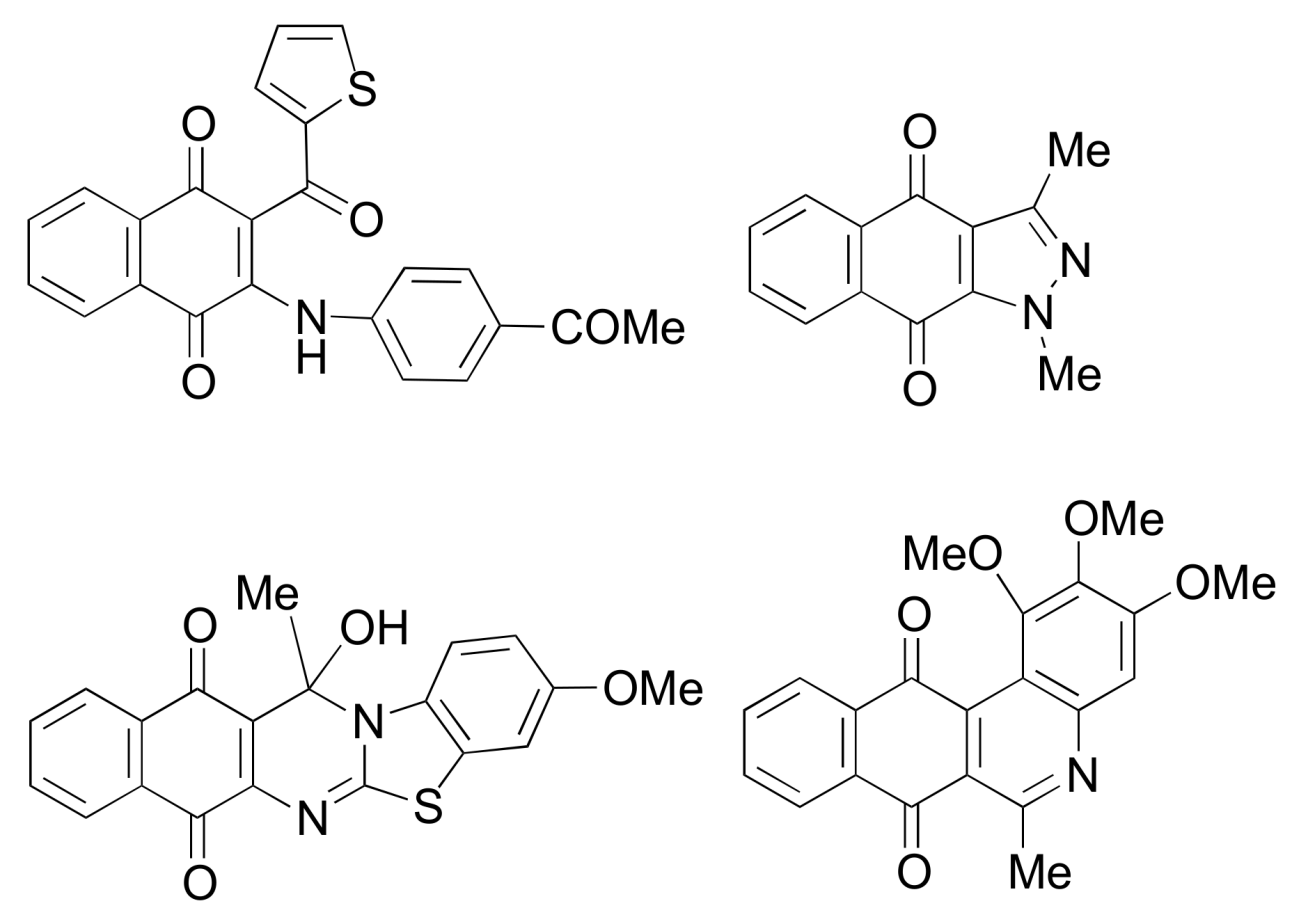
Quinones prepared from acylquinones and nucleophiles.

Acyl-1,4-naphthoquinones, easily achieved from 1,4-quinones and aldehydes, are valuable synthetic precursors of diverse biological active natural and synthetic compounds [1,2,3,4,5,6,7,8,9,10,11,12,13]. Acylnaphthoquinones exhibit outstanding chemical reactions with nucleophiles due to the existence of different electrophilic carbons into these molecules. These features explain the behavior of acylnaphthoquinones to react as monoelectrophiles against aniline derivatives [14,15,16], and as dielectrophiles with ambident nucleophiles such as azaenamines [17], enaminones [18,19], 2-aminobenzothiazoles [20], and trimethoxyaniline [21] to give a variety of carbo- and heterocyclic quinones, such as those outlined in Figure 1.

Older studies reported by Allen and Weiss on the behavior of 2-methoxycarbonyl- and 2-acetyl-1,4-benzoquinone in the Nenitzescu indol synthesis [18], demonstrated that the reaction of the former with ethyl 3-aminocrotonate yield a Michael-type adduct, namely ethyl 2-phenyl-3-aminocrotonate. However, 2-acetyl-1,4-benzoquinone reacts with ethyl 3-aminocrotonate to give the respective dihydroxiisoquinoline derivative through a formal [3+3] process. These facts reveal that ethyl 3-aminocrotonate behaves both as C-nucleophile and as C,N-ambident nucleophile depending upon the nature of the carbonyl substituent bonded at the quinone nucleus. Based on the behavior of 2-acetyl-1,4-benzoquinone with ethyl 3-aminocrotonate, which produces a dihydroxyisoquinoline derivative in a single step, we have reported a general synthetic procedure to prepare diverse cytotoxic isoquinolinequinone-containing compounds from 2-acyl-1,4-quinones and primary enaminones [10,19,22].

Within the framework of target chemotherapeutic agents, a number of studies on the synthesis of cytotoxic carbocyclic quinones linked to amino acid or dipeptide fragments have been reported [23,24,25,26,27]. In this context, we have recently undertaken the synthesis of highly cytotoxic isoquinolinequinone α-amino ester conjugates [28]. In the search for new potential cytotoxic quinones we were interested to evaluate the access to the 1,2-dihydrobenzisoquinolinequinone scaffold through a [3+3] process between acylnaphthoquinones and secondary enaminones derived from α-amino esters. As far as we know, there are no precedents in the literature regarding the assembling into an *N*-heterocyclic scaffold of two biologically relevant naphthoquinones and α-amino acid fragments, through this hypothetical strategy. Herein, we report the reaction of a number of acyl-1,4-naphthoquinones with secondary enaminones derived from aminoethanol and diverse methyl esters of l- and d-α-amino acids. As a result of this study we have developed a convenient access to novel 1,4-naphthoquinones linked to α-amino acid fragments via a vinyl spacer, endowed with in vitro cytotoxic activity on cancer cells.

## 2. Results and Discussion 

**Figure 2:**
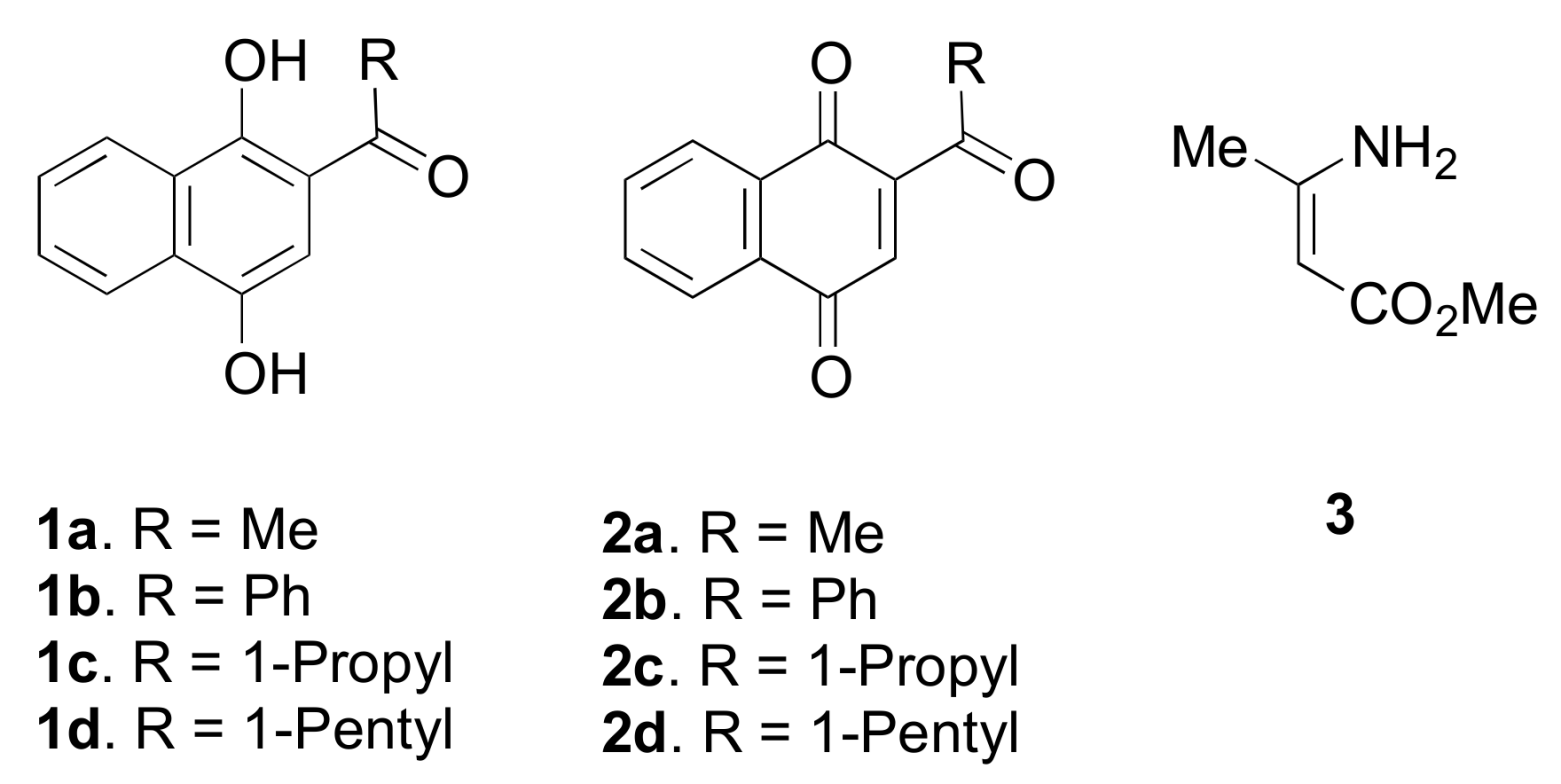
Structure of the quinone and enaminone precursors.

The reactions of the required acylnaphthoquinones **2a**–**d** with the enaminones derived from methyl aminocrotonate **3** (Figure 2) were carried out by means of a one pot procedure, where the electrophiles are in situ generated from their corresponding acylnaphthohydroquinones **1a**–**d** with silver (I) oxide.

**Scheme 1:**
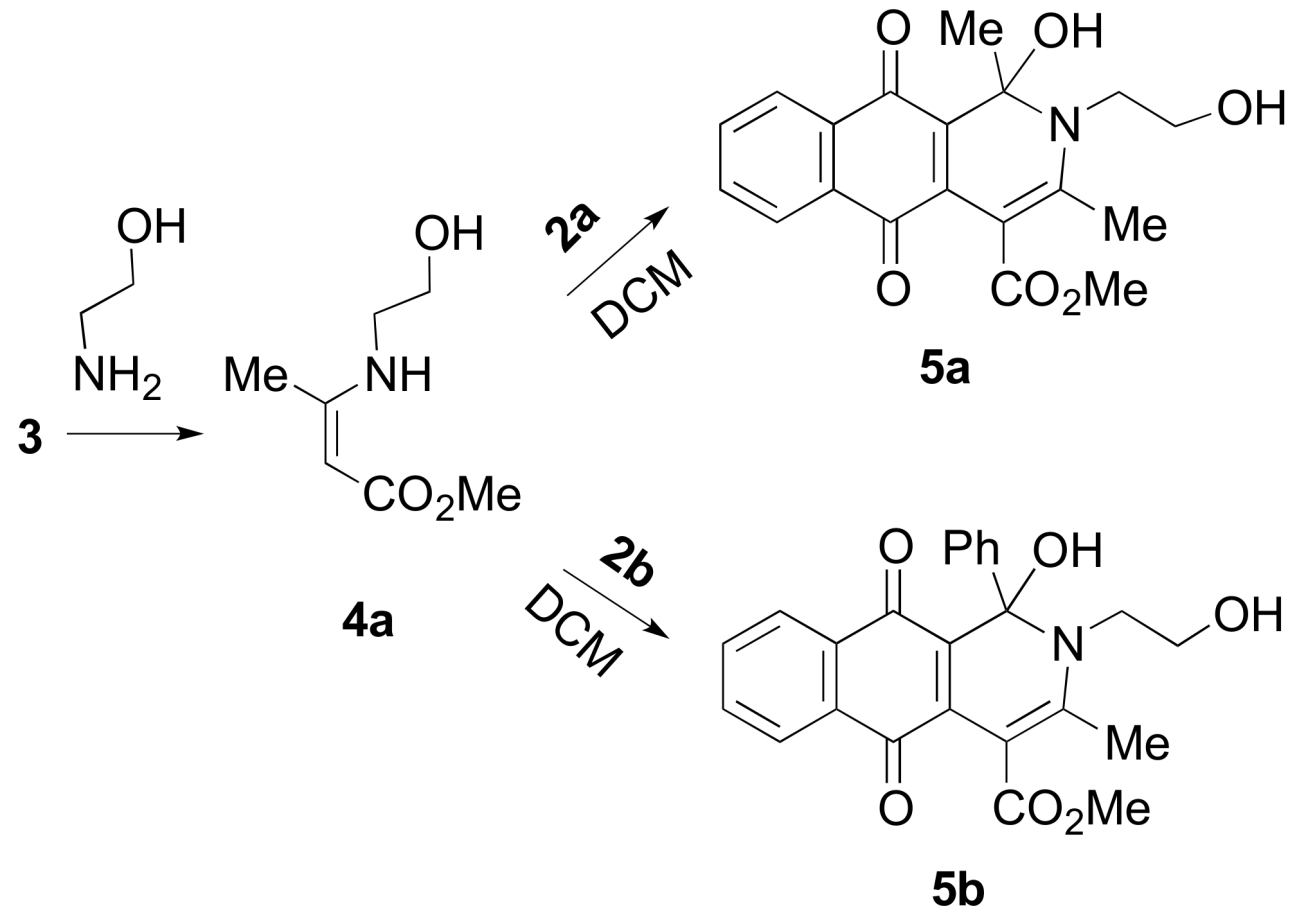
Reaction of acylquinones **2a**,**b** with enaminones **3a**.

Quinones **2a**,**b** were firstly selected to get preliminary insights into their reactivity patterns toward a secondary enaminone such as **4a** (Scheme 1). Compound **4a** was prepared in 81% yield by transamination reaction of methyl 3-aminocrotonate **3** with 2-aminoethanol in methanol, at room temperature. The reactions of acylquinones **2a** and **2b** with enaminone **4a** were carried out at room temperature in dichloromethane (DCM) to produce the corresponding benzisoquinolinequinones **5a** and **5b** in 63 and 72% yield, respectively (Scheme 1). The results confirmed the behavior of the secondary enaminone **4a** to react as a C,N-bidentate nucleophile with the electrophiles **2a** and **2b** to give the respective [3+3] products **5a** and **5b**.

**Scheme 2:**
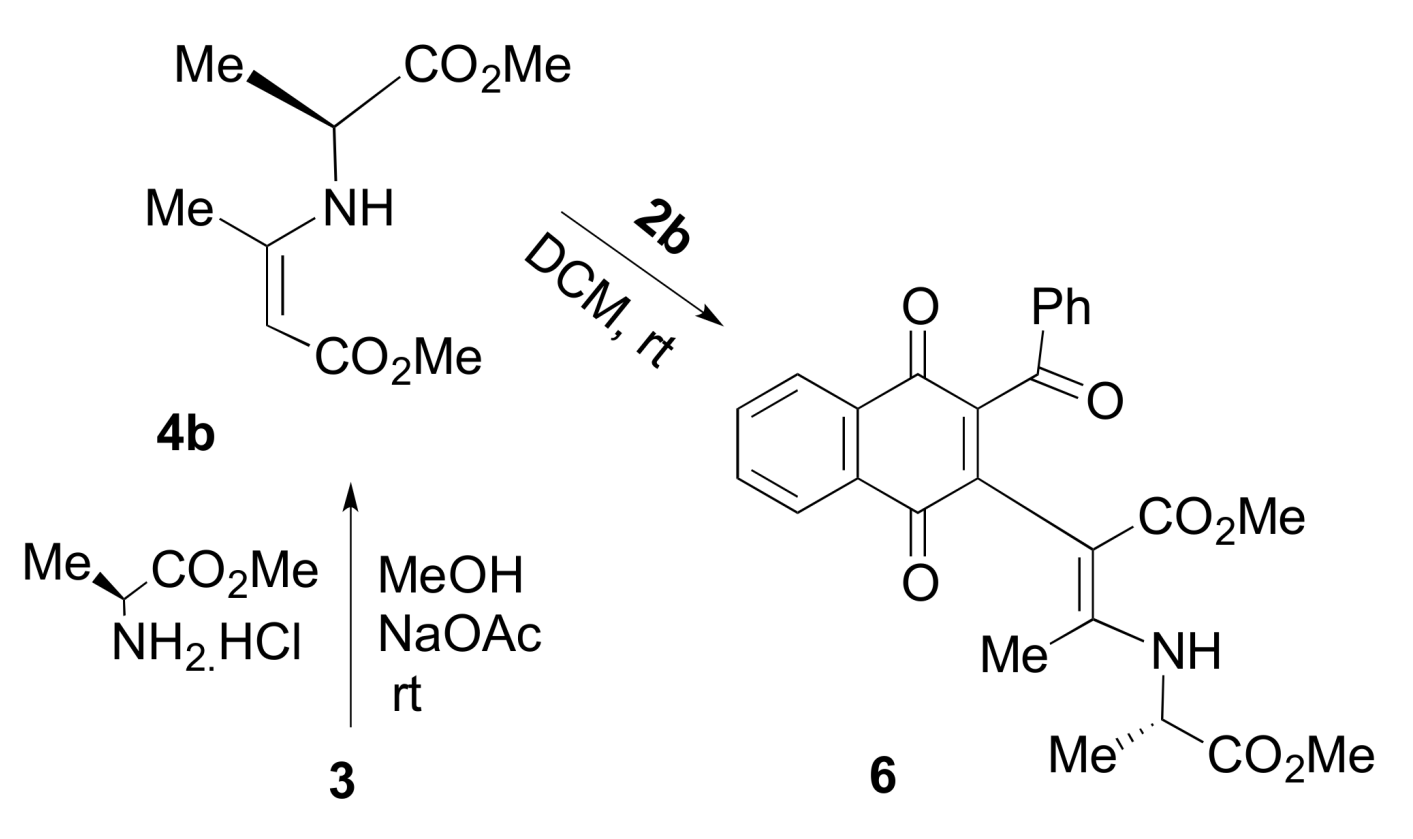
Reaction of quinone **2b** with enaminone **4b**.

Then, we examined the reaction of quinones **2a**,**b** with enaminone **4b**, prepared by reaction of **3** with l-alanine methyl ester (Table 1). The treatment of **2a** with **4b** in methanol, at room temperature provided a complex mixture of products, as was observed by thin layer chromatography (TLC). Interestingly, in the case of quinone **2b**, the treatment with **4b** yield the napthoquinone amino ester conjugate **6** (Scheme 2). This compound was isolated by column chromatography as a 1:1 mixture of two isomers as was evidenced by their ^1^H- and ^13^C-NMR spectra. Structural characterization of **6** was complemented by infrared spectroscopy (IR), bidimensional nuclear magnetic resonance (2D-NMR) and high resolution mass spectroscopy (HRMS).

To the best of our knowledge, the sole precedent regarding the synthesis of quinone amino acid conjugates bonded through a vinyl spacer such as **6** was reported by Bittner et al., employing transamination reactions of diethylamino naphthoquinonic enaminone intermediates with amino acid derivatives [29]. To further evaluate the scope of this interesting one-step formation of an α-amino acid conjugated to a 1,4-napthoquinone core via a vinyl spacer such as **6**, a variety of α-amino acid-derived enaminones **4c**–**g** were prepared from aminocrotonate **3** and a representative number of l- and d-α-amino acid methyl esters (Table 1). The structures of enaminones **4a**–**g** were established by IR, ^1^H-NMR, ^13^C-NMR and HRMS. The Z configuration was assigned for the alkenyl portion of these compounds on the basis of 2D-NMR experiments performed on **4a**,**b**,**g**.

Acylnaphthoquinones **2b**–**d** were reacted with the *N*-substituted aminocrotonate methyl esters **4c**–**g** to give the corresponding napthoquinone amino ester conjugates **7**–**14** in moderate to good yields (Table 2). The structures of the new products **7**–**14** were determined by IR, ^1^H- and ^13^C-NMR and HRMS. As was observed for compound **6**, the ^1^H- and ^13^C-NMR spectra of compounds **7**–**14** revealed that these compounds coexist as two not separable isomers in nearly 1:1 ratio. The Z configuration for **7**–**14** was assigned for the alkenyl portion of these compounds on the basis of 2D-NMR experiments performed in compounds **6**–**8**.

**Figure 3:**
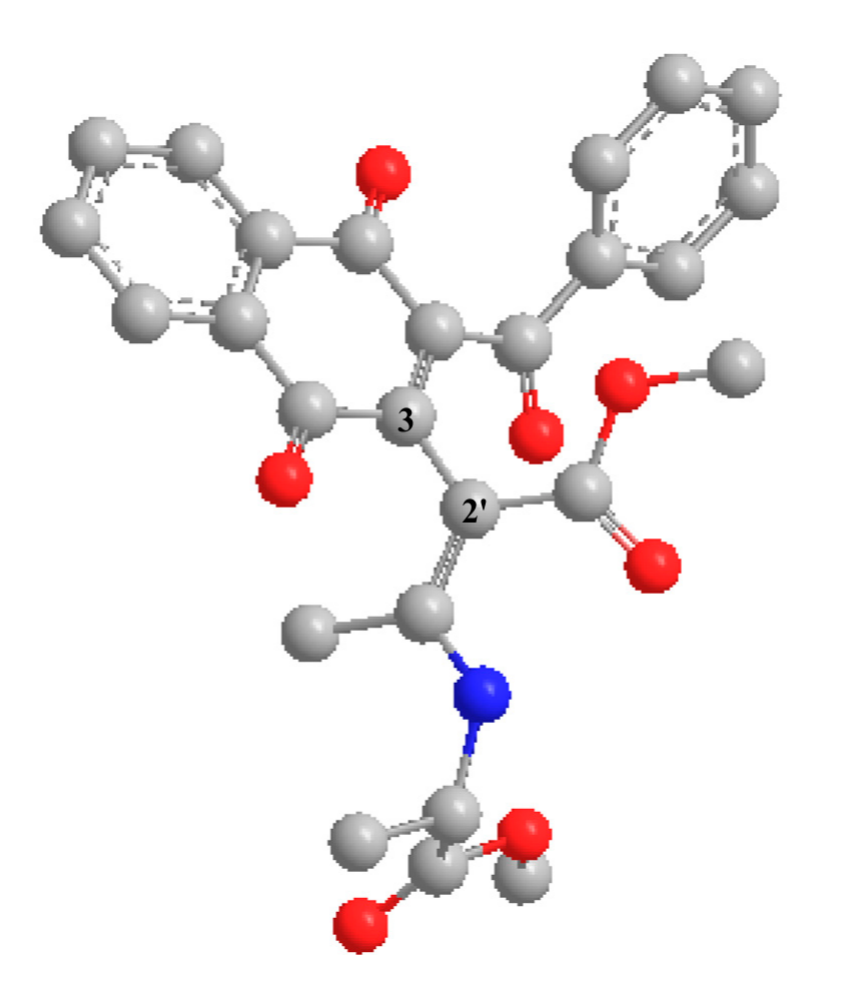
Molecular model of one conformational isomer of **6**.

It is noteworthy that compounds **6**–**14** exhibit homogeneous properties in terms of their melting points and TLC. Based on these facts, it is probable that compounds **6**–**14** exist as a mixture of two conformational isomers arising from hindered internal molecular rotation. Inspection of one minimal energy conformation of compounds **6**, represented in ball and stick mode, shows that rotation of the substituents linked to the quinone double bond is strongly hindered, in particular about the 3-2’ C-C bond (Figure 3).

To provide further proofs of the existence of conformational isomerism in the members of the series **6**–**14**, compounds **7** and **9** were subject to ^1^H-NMR coalescence experiments in DMSO-*d*_6_ at temperatures over 25.6 °C. In the ^1^H-NMR spectrum of **7** the signals of the amine protons appear downfield as two doublets at δ 10.09 and 10.20. As the temperature increased, the two signals broadened, and coalesced at ~348 K. In the case of compound **9**, it was observed that the signals of the amine and methine protons at δ 9.38/9.43 and 4.65/4.76 coalesced at ~378 K. Therefore, these rather high coalescence temperatures are consistent with the doubling of signals observed in the ^1^H- and ^13^C-NMR spectra of compounds **6**–**14** at room temperature.

The results of the reactions of acylquinones **2b**–**d** with **4b**–**g** revealed that the secondary enaminones behave either as C-unidentate or as a C,N-bidentate nucleophile depending upon the structure of the nitrogen substituents bonded at the enaminone C,C double bond. It is reasonable to assume that the [3+3] process, observed in the reactions of quinones **2a**,**b** with enaminone **4a**, proceed through a Michael adduct intermediate that undergoes a 6-exo trig closure. Based on this scenario, the lack of cyclisation of the Michael adduct intermediates derived from the amino esters-enaminones **4b**–**g**, may be attributed to steric factors.

The series of naphthoquinone amino ester conjugates **6**–**14** were evaluated in vitro for their cytotoxic activity against normal human lung fibroblast (MRC-5) and three human cancer cells lines: human gastric adenocarcinoma (AGS), human lung cancer (SK-MES-1) and human bladder carcinoma (J82), in 72 h drugs exposure assays (Table 3). The cytotoxic activity of the new compounds was measured using conventional microculture tetrazolium reduction assays [30]. Cytotoxic activities of the compounds are expressed in terms of IC_50_. Etoposide, a clinically used anticancer agent, was taken as a positive control. The cytotoxic activity data are summarized in Table 3.

Table 3 shows moderate cytotoxic activities for **6**–**14**, in the range IC_50_ = 4.5–53.4 μM, lower than those displayed by the drug etoposide. Compound **14** appears as the most potent member of the series on human lung and bladder carcinoma cell lines (IC_50_: 5.5 and 4.5 μM) and with cytotoxicity ten times lower than the drug etoposide on normal human lung fibroblasts cells.

## 3. Materials and Methods 

### 3.1. General 

All solvents and reagents were purchased from different companies such as Aldrich (St. Louis, MO, USA) and Merck (Darmstadt, Germany) and were used as supplied. Melting points were determined on a Stuart Scientific SMP3 (Bibby Sterilin Ltd., Staffordshire, UK) apparatus and are uncorrected. The IR spectra were recorded on a FT IR Bruker spectrophotometer; (model Vector 22 Bruker, Rheinstetten, Germany), using KBr disks, and the wave numbers are given in cm^−1^. ^1^H- and ^13^C-NMR spectra were recorded on Bruker Avance-400 instrument (Bruker, Ettlingen, Germany) in CDCl_3_ at 400 and 100 MHz, respectively. Chemical shifts are expressed in ppm downfield relative to tetramethylsilane and the coupling constants (*J*) are reported in Hertz. Data for ^1^H-NMR spectra are reported as follows: s = singlet, br s = broad singlet, d = doublet, m = multiplet and the coupling constants (*J*) in Hz. Bidimensional NMR techniques (HMBC and NOESY) and distortionless enhancement by polarisation transfer (DEPT) were used for signal and E/Z configuration assignments. The ^1^H-NMR coalescence experiments were recorded in DMSO-*d*_6_ solutions on the Bruker spectrometer operating at 400 MHz equipped with the 5 mm PAQNP probe. HRMS-ESI were carried out by using a Thermo Scientific Exactive Plus Orbitrap spectrometer with a constant nebulizer temperature of 250 °C. The experiments were performed in positive ion mode, with a scan range of *m*/*z* 100–300. All fragment ions were assigned by accurate mass measurements at high resolution (resolving power: 140,000 FWHM). The samples were infused directly into the electrospray ionization source (ESI) using a syringe pump at flow rates of 5 μL min^−1^. Optical rotations were obtained for CHCl_3_ solutions in a Polarimeter instrument (Optical Activity Ltd., Cambridgeshire, UK) in a 1 dm cell and their concentrations are expressed in g per mL. Silica gel Merck 60 (70–230 mesh, from Merck, Darmstadt, Germany) was used for preparative column chromatography and TLC aluminum foil 60F254 for analytical thin layer chromatography (TLC).

*Methyl (Z)-3-[(2-hydroxyethyl)amino]-but-2-enoate* (**4a**). A solution of methyl 3-aminocrotonate **3** (100 mg, 0.87 mmol), 2-aminoethanol (1.04 mmol) and methanol (10 mL) was stirred at r.t. for 3 h. After completion of the reaction as indicated by TLC, the solvent was removed under reduced pressure and the residue was purified by chromatography over silica gel (CH_2_Cl_2_) to yield pure enaminone **4a** (112 mg, 0.70 mmol, 81%) as yellow oil; IR ν_max_: 3340, 2947 and 1636 cm^−1^; ^1^H-NMR: δ 1.99 (s, 3H, CH_3_), 2.87 (br s, 1H, OH), 3.34 (m, 2H, CH_2_OH), 3.58 (s, 3H, CH_3_), 3.71 (m, 2H, CH_2_NH), 4.45 (s, 1H, CH), 8.61 (br s, 1H, NH), ^13^C-NMR: δ 19.7, 45.2, 50.1, 61.9, 82.3, 162.4, 171.2. HRMS (M^+^): *m*/*z* calcd. for C_7_H_13_NO_3_:159.0895; found: 159.0964.

### 3.2. Preparation of Enaminone-Amino Acid Derivatives. General Procedure

Suspensions of methyl 3-aminocrotonate **3** (1 equiv.), l- or d-α-amino acid methyl esters hydrochloride (1.2 equiv.) and NaOAc (1.2 equiv.) in methanol (15 mL) were stirred at room temperature until completion of the reaction as indicated by TLC. The solvents were removed under reduced pressure and the residues purified by column chromatography over silica gel (CH_2_Cl_2_) to yield the corresponding enaminones **4b**–**g**.

*Methyl (S,Z)-3-[(1-methoxy-1-oxopropan-2-yl)amino]-but-2-enoate* (**4b**). Prepared from **3** (100 mg, 0.87 mmol) and l-alanine methyl ester hydrochloride (2 h, 125 mg, 0.62 mmol, 71%): yellow oil, IR ν_max_: 2988, 1741 and 1657 cm^−1^; ^1^H-NMR: δ 1.48 (d, 3H, *J* = 7.2 Hz, CH_3_), 1.90 (s, 3H, CH_3_), 3.64 (s, 3H, CO_2_CH_3_), 3.76 (s, 3H, CO_2_CH_3_), 4.21 (m, 1H, CH), 4.53 (s, 1H, CH), 8.76 (br s, 1H, NH). ^13^C-NMR: δ 19.3, 19.4, 50.1, 51.3, 52.5, 84.0, 160.2, 170.7, 173.3. HRMS (M^+^): *m*/*z* calcd. for C_9_H_15_NO_4_: 201.1001; found: 201.1072. ${\left[\alpha \right]}_{D}^{22}$ = +16.47 (c = 3.46, CHCl_3_).

*Methyl (R,Z)-3-[(1-methoxy-1-oxopropan-2-yl)amino]-but-2-enoate* (**4c**). Prepared from **3** (100 mg, 0.87 mmol) and d-alanine methyl ester hydrochloride (1.2 h, 148 mg, 0.74 mmol, 85%): yellow oil, IR ν_max_: 2988, 1741 and 1657 cm^−1^; ^1^H-NMR: δ 1.48 (d, 3H, *J* = 7.2 Hz, CH_3_), 1.90 (s, 3H, CH_3_), 3.64 (s, 3H, CO_2_CH_3_), 3.76 (s, 3H, CO_2_CH_3_), 4.21 (m, 1H, CH), 4.53 (s, 1H, CH), 8.76 (br s, 1H, NH). ^13^C-NMR: δ 19.3, 19.4, 50.1, 51.3, 52.5, 84.04, 160.2, 170.7, 173.3. HRMS (M^+^): *m*/*z* calcd. for C_9_H_15_NO_4_: 201.1001; found: 201.1069. ${\left[\alpha \right]}_{D}^{22}$ = −15.93 (c = 3.39, CHCl_3_).

*Methyl (Z)-(4-methoxy-4-oxobut-2-en-2-yl)-l-leucinate* (**4d**). Prepared from **3** (300 mg, 2.60 mmol) and l-leucine methyl ester hydrochloride (2 h, 507 mg, 2.09 mmol, 80%): yellow oil, IR ν_max_: 2986, 1739 and 1660 cm^−1^; ^1^H-NMR: δ 0.92 (d, 3H, *J* = 6.4 Hz, CH_3_), 0.96 (d, 3H, *J* = 6.4 Hz, CH_3_), 1.25 (m, 1H, CH_2_), 1.68 (m, 1H, CH_2_), 1.76 (m, 1H, CH), 1.88 (s, 3H, CH_3_), 3.63 (s, 3H, CO_2_CH_3_), 3.73 (s, 3H, CO_2_CH_3_), 4.11 (m, 1H, CH), 4.53 (s, 1H, CH), 8.68 (d, 1H, *J* = 8.0 Hz, NH). ^13^C-NMR: δ 19.6, 21.9, 22.94, 24.7, 42.2, 50.2, 52.5, 54.6, 84.3, 160.6, 170.8, 173.4. HRMS (M^+^): *m*/*z* calcd. for C_12_H_21_NO_4_: 243.1471; found: 243.1579. ${\left[\alpha \right]}_{D}^{22}$ = +11.39 (c = 2.02, CHCl_3_).

*Methyl (S,Z)-3-[(1-methoxy-1-oxo-3-phenylpropan-2-yl)amino]-but-2-enoate* (**4e**). Prepared from **3** (100 mg, 0.87 mmol) and l-phenylalanine methyl ester hydrochloride (3 h, 200 mg, 0.72 mmol, 83%): white solid, m.p.: 75–77 °C; IR ν_max_: 2986, 1735 and 1685 cm^−1^; ^1^H-NMR: δ 1.62 (s, 3H, CH_3_), 2.98 (dd, 1H, *J* = 8.8, 13.6 Hz, CH_2_), 3.15 (dd, 1H, *J* = 5.2, 13.6 Hz, CH_2_), 3.63 (s, 3H, CO_2_CH_3_), 3.71 (s, 3H, CO_2_CH_3_), 4.11 (m, 1H, CH), 4.45 (s, 1H, CH), 7.20 (m, 2H, arom.), 7.26 (m, 3H, arom.), 8.90 (d, 1H, *J* = 9.2 Hz, NH). ^13^C-NMR: δ 19.2, 40.3, 50.1, 52.4, 57.9, 84.3, 127.1, 128.6 (2C), 129.3 (2C), 136.3, 159.9, 170.5, 171.9. HRMS (M^+^): *m*/*z* calcd. for C_15_H_19_NO_4_: 277.1314; found: 277.1386. ${\left[\alpha \right]}_{D}^{22}$ = +15.22 (c = 2.46, CHCl_3_).

*Methyl (R,Z)-3-[(1-methoxy-1-oxo-3-phenylpropan-2-yl)amino]-but-2-enoate* (**4f**). Prepared from **3** (100 mg, 0.87 mmol) and D-phenylalanine methyl ester hydrochloride (3 h, 183 mg, 0.67 mmol, 76%): white solid, m.p.: 76–79 °C; IR ν_max_: 2986, 1735 and 1685 cm^−1^; ^1^H-NMR: δ 1.62 (s, 3H, CH_3_), 2.98 (dd, 1H, *J* = 8.8, 13.6 Hz, CH_2_), 3.15 (dd, 1H, *J* = 5.2, 13.6 Hz, CH_2_), 3.63 (s, 3H, CO_2_CH_3_), 3.71 (s, 3H, CO_2_CH_3_), 4.11 (m, 1H, CH), 4.45 (s, 1H, CH), 7.20 (m, 2H, arom.), 7.26 (m, 3H, arom.), 8.90 (d, 1H, *J* = 9.2 Hz, NH). ^13^C-NMR: δ 19.2, 40.3, 50.1, 52.4, 57.9, 84.3, 127.1, 128.6 (2C), 129.3 (2C), 136.3, 159.9, 170.5, 171.9. HRMS (M^+^): *m*/*z* calcd. for C_15_H_19_NO_4_: 277.1314; found: 277.1326. ${\left[\alpha \right]}_{D}^{22}$ = −14.16 (c = 2.33, CHCl_3_).

*Methyl (S,Z)-3-[(3-(1H-indol-3-yl)-1-methoxy-1-oxopropan-2-yl)amino]-but-2-enoate* (**4g**). Prepared from **3** (100 mg, 0.87 mmol) and l-tryptophan methyl ester hydrochloride (3.5 h, 231 mg, 0.73 mmol, 84%): white solid, m.p.: 96–98 °C; IR ν_max_: 2989, 1737 and 1682 cm^−1^; ^1^H-NMR: δ 1.65 (s, 3H, CH_3_), 3.19 (dd, 1H, *J* = 7.6, 14.8 Hz, CH_2_), 3.30 (dd, 1H, *J* = 4.8, 14.4 Hz, CH_2_), 3.61 (s, 3H, CO_2_CH_3_), 3.65 (s, 3H, CO_2_CH_3_), 4.41 (m, 1H, CH), 4.44 (s, 1H, CH), 7.02 (s, 1H, indoyl), 7.10 (m, 2H, arom.), 7.26 (d, 1H, *J* = 8.0 Hz, arom.), 7.54 (d, 1H, *J* = 7.6 Hz, arom.), 8.65 (br s, 1H, N-indoyl), 8.95 (d, 1H, *J* = 9.2 Hz, NH). ^13^C-NMR: δ 19.3, 29.8, 50.1, 52.4, 56.6, 84.0, 109.4, 111.5, 118.2, 119.3, 121.9, 123.8, 127.0, 136.2, 160.34, 170.7, 172.5. HRMS (M^+^): *m*/*z* calcd. for C_17_H_20_N_2_O_4_: 316.1423; found: 316.1536. ${\left[\alpha \right]}_{D}^{22}$ = +14.21 (c = 1.97, CHCl_3_).

### 3.3. Preparation of Compounds ***5a,b*** and ***6***–***14***. General Procedure

Suspensions of acylnaphthohydroquinones **1a**–**d** (1 equiv.), enaminones **4a**–**g** (1.2 equiv.), Ag_2_O (2 equiv.) and MgSO_4_ (0.5 g) in CH_2_Cl_2_ (25 mL) were stirred at room temperature until completion of the reaction as indicated by TLC. The mixtures were filtered, the solids washed with CH_2_Cl_2_ and the solvent removed under reduced pressure; the residues were purified by chromatography over silica gel (90:10 CH_2_Cl_2_/EtOAc) to yield the corresponding naphthoquinone derivatives.

*Methyl 1-hydroxy-2-(2-hydroxyethyl)-1,3-dimethyl-5,10-dioxo-1,2,5,10-tetrahydrobenzo[g]isoquinoline-4-carboxylate* (**5a**). Prepared from **1a** (100 mg, 0.49 mmol) and **4a** (2 h, 112 mg, 0.31 mmol, 64%): purple solid, m.p.: 215–217 °C; IR ν_max_: 3424, 2948, 1721, 1671 and 1498 cm^−1^; ^1^H-NMR: δ 1.44 (s, 3H, CH_3_), 2.27 (s, 1H, OH), 2.76 (s, 3H, CH_3_), 3.74 (m, 1H, CH_2_OH), 3.86 (m, 1H, CH_2_OH), 3.95 (s, 4H, CO_2_CH_3_ and OH), 4.04 (m, 1H, CH_2_NH), 4.27 (m, 1H, CH_2_NH), 7.63 (dd, 1H, *J* = 7.2 and 8.0 Hz, arom.), 7.72 (dd, 1H, *J* = 6.8 and 8.4 Hz, arom.), 8.12 (d, 1H, *J* = 8.0 Hz, arom.), 8.27 (d, 1H, *J* = 8.0 Hz, arom.). ^13^C-NMR: δ 19.6, 22.6, 46.5, 52.7, 64.4, 92.5, 105.4, 119.7, 126.9, 127.0, 128.5, 131.1, 133.3, 134.4, 137.1, 155.0, 167.10, 179.3, 182.2. HRMS (M^+^): *m*/*z* calcd. for C_19_H_19_NO_6_: 357.1212; found: 357.1225.

*Methyl 1-hydroxy-2-(2-hydroxyethyl)-3-methyl-5,10-dioxo-1-phenyl-1,2,5,10-tetrahydrobenzo[g] isoquinoline-4-carboxylate* (**5b**). Prepared from **1b** (100 mg, 0.38 mmol) and **4a** (1.5 h, 115 mg, 0.27 mmol, 72%): red solid, m.p.: 236.5–238.5 °C; IR ν_max_: 3356, 2948, 1735, 1678 and 1435 cm^−1^; ^1^H-NMR: δ 1.60 (s, 3H, CH_3_), 3.32 (m, 1H, CH_2_OH), 3.67 (m, 1H, CH_2_OH), 3.79 (m, 2H, CH_2_NH and OH), 3.99 (s, 3H, CO_2_CH_3_), 4.12 (m, 2H, CH_2_NH and OH), 7.27 (d, 1H, *J* = 7.2 Hz, arom.), 7.39 (dd, 1H, *J* = 7.2 and 7.6 Hz, arom.), 7.54 (m, 3H, arom.), 7.66 (m, 2H, arom.), 8.13 (d, 1H, *J* = 7.6 Hz, arom.), 8.17 (d, 1H, *J* = 7.6 Hz, arom.). ^13^C-NMR: δ 23.6, 49.5, 52.7, 65.5, 92.5, 106.0, 120.4, 126.9, 127.0, 128.5, 128.8, 128.9, 129.0, 129.1, 130.5, 132.3, 133.6, 134.4, 134.5, 136.7, 155.0, 166.8, 178.6, 182.1. HRMS (M^+^): *m*/*z* calcd. for C_24_H_21_NO_6_: 419.1369; found: 419.1345.

*Methyl (S,Z)-2-(3-benzoyl-1,4-dioxo-1,4-dihydronaphthalen-2-yl)-3-[(1-methoxy-1-oxopropan-2-yl) amino]-but-2-enoate* (**6**). Prepared from **1b** (100 mg, 0.38 mmol) and **4b** (1.5 h, 102 mg, 0.22 mmol, 58%); purple solid; isomers proportion ~45:55; m.p.: 217–218 °C; IR ν_max_: 2988, 1738, 1681, 1664 and 1650 cm^−1^; ^1^H-NMR: δ 1.40 (d, 3H, *J* = 9.2 Hz, CH_3_), 1.46 (d, 3H, *J* = 9.2 Hz, CH_3_), 1.64 (s, 6H, CH_3_), 3.45 (s, 6H, CO_2_CH_3_), 3.75 (s, 3H, CO_2_CH_3_), 3.76 (s, 3H, CO_2_CH_3_), 4.19 (m, 2H, CH), 7.40 (m, 4H, arom.), 7.56 (m, 2H, arom.), 7.79–7.87 (m, 8H, arom.), 8.13 (m, 2H, arom.), 8.19 (m, 2H, arom.), 9.57 (d, 1H, *J* = 10.4 Hz, NH), 9.68 (d, 1H, *J* = 11.2 Hz, NH). ^13^C-NMR: δ 17.5, 17.6, 19.3, 19.7, 50.7, 51.8, 52.7, 52.8, 87.6, 87.7, 126.5, 126.60, 127.1, 127.2, 128.5, 128.6, 129.1, 129.2, 132.0, 132.1, 132.4, 132.5, 134.1, 134.2, 134.1, 134.2, 134.3, 135.7, 146.0, 146.1, 160.6, 160.7, 167.7, 172.59, 172.7, 184.1, 184.6, 184.9, 193.0, 193.2. HRMS (M^+^): *m*/*z* calcd. for C_26_H_23_NO_7_: 461.1474; found: 463.1547.

*Methyl (R,Z)-2-(3-benzoyl-1,4-dioxo-1,4-dihydronaphthalen-2-yl)-3-[(1-methoxy-1-oxopropan-2-yl)amino]-but-2-enoate* (**7**). Prepared from **1b** (100 mg, 0.38 mmol) and **4c** (1.5 h, 91.52 mg, 0.20 mmol, 52%); isomers proportion ~47:53; purple oil; IR ν_max_: 2988, 1738, 1681, 1664 and 1650 cm^−1^; ^1^H-NMR: δ 1.40 (d, 3H, *J* = 9.2 Hz, CH_3_), 1.45 (d, 3H, *J* = 9.6 Hz, CH_3_), 1.64 (s, 6H, CH_3_), 3.45 (s, 6H, CO_2_CH_3_), 3.75 (s, 3H, CO_2_CH_3_), 3.76 (s, 3H, CO_2_CH_3_), 4.15 (m, 2H, CH), 7.40 (m, 4H, arom.), 7.56 (m, 2H, arom.), 7.79–7.87 (m, 8H, arom.), 8.13 (m, 2H, arom.), 8.18 (m, 2H, arom.), 9.57 (d, 1H, *J* = 10.8 Hz, NH), 9.68 (d, 1H, *J* = 11.2 Hz, NH). ^13^C-NMR: δ 17.5, 17.6, 19.3, 19.7, 50.7, 51.7, 52.7, 52.8, 87.6, 87.7, 126.5, 126.6, 127.1, 127.2, 128.5, 128.6, 129.1, 129.2, 132.0, 132.1, 132.4, 132.5, 134.1, 134.2, 134.1, 134.2, 134.3, 135.7, 145.9, 146.0, 160.6, 160.7, 167.7, 172.6, 172.7, 184.1, 184.6, 184.9, 192.9, 193.2. HRMS (M^+^): *m*/*z* calcd. for C_26_H_23_NO_7_: 461.1474; found: 461.1540.

*Methyl (Z)-[3-(3-benzoyl-1,4-dioxo-1,4-dihydronaphthalen-2-yl)-4-methoxy-4-oxobut-2-en-2-yl]-l-leucinate* (**8**). Prepared from **1b** (100 mg, 0.38 mmol) and **4d** (2 h, 80.63 mg, 0.16 mmol, 42%); isomers proportion ~47:53; red solid; m.p.: 222.5–224.5 °C; IR ν_max_: 2990, 1740, 1681, 1662, 1645 cm^−1^; ^1^H-NMR: δ 0.88 (m, 6H, CH_3_), 0.91 (d, 3H, *J* = 6.4 Hz, CH_3_), 0.94 (d, 3H, *J* = 6.4 Hz, CH_3_), 1.46–1.78 (m, 6H, CH and CH_2_), 1.83(s, 6H, CH_3_), 3.45 (s, 6H, CO_2_CH_3_), 3.74 (s, 6H, CO_2_CH_3_), 4.11 (m, 2H, CH), 7.40 (m, 4H, arom.), 7.55 (m, 2H, arom.), 7.78–7.86 (m, 8H, arom.), 8.13 (m, 2H, arom.), 8.19 (m, 2H, arom.), 9.46 (d, 1H, *J* = 8.0 Hz, N-H), 9.55 (d, 1H, *J* = 8.8 Hz, N-H). ^13^C-NMR: δ 17.6, 17.7, 22.0, 22.1, 22.8, 22.9, 24.6, 24.7, 42.0, 42.3, 50.6, 50.7, 52.5, 52.6, 54.9, 87.8, 87.9, 126.5, 127.1, 127.2, 128.5, 129.1, 129.2, 132.1, 132.4, 132.5, 134.0, 134.1, 134.2, 134.3, 135.7, 135.8, 143.8, 143.91, 145.9, 146.0, 160.7, 161.1, 167.6, 167.7, 172.4, 172.5, 183.9, 184.1, 184.5, 184.8, 192.8, 192.9. HRMS (M^+^): *m*/*z* calcd. for C_29_H_29_NO_7_: 503.1944; found: 503.2005.

*Methyl (S,Z)-2-(3-benzoyl-1,4-dioxo-1,4-dihydronaphthalen-2-yl)-3-[(1-methoxy-1-oxo-3-phenylpropan-2-yl)amino]-but-2-enoate* (**9**). Prepared from **1b** (100 mg, 0.38 mmol) and **4e** (2.5 h, 133.2 mg, 0.25 mmol, 65%); isomers proportion ~50:50; purple solid; m.p.: 176.5–178 °C; IR ν_max_: 2992, 1735, 1688, 1660 and 1655 cm^−1^; ^1^H-NMR: δ 1.59 (s, 3H, CH_3_), 1.76 (s, 3H, CH_3_), 3.00 (m, 3H, CH_2_), 3.12 (dd, 1H, *J* = 5.2, 13.6 Hz, CH_2_), 3.43 (s, 3H, CO_2_CH_3_), 3.45 (s, 3H, CO_2_CH_3_), 3.69 (s, 3H, CO_2_CH_3_), 3.70 (s, 3H, CO_2_CH_3_), 4.30 (m, 1H, CH), 4.36 (m, 1H, CH), 6.97 (d, 2H, *J* = 6.8 Hz, arom.), 7.18–7.27 (m, 6H, arom.), 7.32 (m, 2H, arom.), 7.37–7.44 (m, 4H, arom.), 7.56 (m, 2H, arom.), 7.77–7.86 (m, 8H, arom.), 8.11 (m, 2H, arom.), 8.18 (m, 2H, arom.), 9.74 (t, 2H, *J* = 9.4 Hz, NH). ^13^C-NMR: δ 17.3, 7.5, 40.3, 50.6, 52.6, 57.8, 58.2, 88.0, 126.5, 127.1, 127.2, 127.3, 127.4, 128.5, 128.6, 128.7, 128.9, 129.1, 129.2, 129.3, 129.5, 132.1, 132.3, 132.4, 134.01, 134.02, 134.08, 134.13, 134.25, 135.5, 135.6, 135.7, 135.8, 143.8, 143.9, 145.9, 146.0, 160.0, 160.6, 167.5, 167.6, 171.1, 171.4, 184.0, 184.1, 184.5, 184.6, 192.8, 192.9. HRMS (M^+^): *m*/*z* calcd. for C_32_H_27_NO_7_: 537.1788; found: 537.1852.

*Methyl (R,Z)-2-(3-benzoyl-1,4-dioxo-1,4-dihydronaphthalen-2-yl)-3-[(1-methoxy-1-oxo-3-phenylpropan-2-yl)amino]-but-2-enoate* (**10**). Prepared from **1b** (100 mg, 0.38 mmol) and **4f** (3 h, 118.8 mg, 0.22 mmol, 58%); isomers proportion ~50:50; red solid; m.p.: 154.5–156.5 °C; IR ν_max_: 2992, 1735, 1688, 1660 and 1655 cm^−1^; ^1^H-NMR: δ 1.60 (s, 3H, CH_3_), 1.76 (s, 3H, CH_3_), 3.00 (m, 3H, CH_2_), 3.11 (dd, 1H, *J* = 5.2, 13.6 Hz, CH_2_), 3.43 (s, 3H, CO_2_CH_3_), 3.45 (s, 3H, CO_2_CH_3_), 3.68 (s, 3H, CO_2_CH_3_), 3.69 (s, 3H, CO_2_CH_3_), 4.31 (m, 1H, CH), 4.37 (m, 1H, CH), 6.97 (d, 2H, *J* = 6.8 Hz, arom.), 7.17–7.24 (m, 6H, arom.), 7.31 (m, 2H, arom.), 7.37–7.43 (m, 4H, arom.), 7.55 (m, 2H, arom.), 7.76–785 (m, 8H, arom.), 8.11 (m, 2H, arom.), 8.17 (m, 2H, arom.), 9.74 (t, 2H, *J* = 9.2 Hz, NH). ^13^C-NMR: δ 17.3, 17.4, 40.2, 50.57, 52.5, 57.7, 58.1, 87.9, 88.0, 126.4, 127.0, 127.1, 127.22, 127.3, 128.45, 128.5, 128.6, 128.8, 129.0, 129.1, 129.2, 129.4, 132.0, 132.3, 132.3, 133.9, 134.0, 134.05, 134.1, 134.2, 135.4, 135.6, 135.7, 135.8, 143.7, 143.8, 145.8, 145.9, 159.95, 160.5, 167.4, 167.5, 171.0, 171.3, 183.9, 184.0, 184.4, 184.5, 192.8, 192.9. HRMS (M^+^): *m*/*z* calcd. for C_32_H_27_NO_7_: 537.1788; found: 537.1861.

*Methyl (S,Z)-3-[(3-(1H-indol-3-yl)-1-methoxy-1-oxopropan-2-yl)amino]-2-(3-benzoyl-1,4-dioxo-1,4-dihydronaphthalen-2-yl)-but-2-enoate* (**11**). Prepared from **1b** (100 mg, 0.38 mmol) and **4g** (2.5 h, 155.9 mg, 0.27 mmol, 71%); isomers proportion ~45:55; purple solid; m.p.: 230.5–232.5. °C; IR ν_max_: 2990, 1735, 1697, 1670 and 1650 cm^−1^; ^1^H-NMR: δ 1.55 (s, 3H, CH_3_), 1.58 (s, 3H, CH_3_), 3.08–3.18 (m, 3H, CH_2_), 3.27 (dd, 1H, *J* = 5.2, 14.4 Hz, CH_2_), 3.39 (s, 3H, CO_2_CH_3_), 3.45 (s, 3H, CO_2_CH_3_), 3.64 (s, 3H, CO_2_CH_3_), 3.66 (s, 3H, CO_2_CH_3_), 4.39 (m, 2H, CH), 6.77 (s, 1H, CH), 7.0–7.15 (m, 5H, arom.), 7.24–7.41 (m, 4H, arom.), 7.46–7.57 (m, 6H, arom.), 7.76 (m, 4H, arom.), 7.82 (d, 2H, *J* = 7.6 Hz, arom.), 7.87 (d, 2H, *J* = 7.6 Hz, arom.), 8.09 (d, 2H, *J* = 7.6 Hz, arom.), 8.15 (m, 2H, arom.), 8.48 (br s, 1H, NH), 8.52 (br s, 1H, NH), 9.76 (d, 1H, *J* = 8.8 Hz, NH), 9.93 (d, 1H, *J* = 8.8 Hz, NH). ^13^C-NMR: δ 17.3, 17.4, 29.9, 30.2, 50.5, 50.6, 52.5, 52.6, 56.9, 87.3, 87.5, 109.0, 109.1, 111.4, 111.5, 118.1, 118.2, 119.4, 119.5, 122.0, 122.1, 123.8, 126.4, 126.5, 126.9, 127.0, 127.01, 127.1, 128.4, 128.6, 129.0, 129.1, 131.9, 132.0, 132.2, 132.3, 134.0, 134.15, 134.20, 134.25, 135.6, 136.1, 136.0, 143.8, 144.1, 145.9, 160.6, 160.9, 167.5, 167.6, 171.2, 171.6, 184.0, 184.1, 184.5, 184.8, 192.8, 193.1. HRMS (M^+^): *m*/*z* calcd. for C_34_H_28_N_2_O_7_: 576.1897; found: 576.1956.

*Methyl (R,Z)-2-(3-butyryl-1,4-dioxo-1,4-dihydronaphthalen-2-yl)-3-[(1-methoxy-1-oxo-3-phenylpropan-2-yl)amino]-but-2-enoate* (**12**). Prepared from **1c** (200 mg, 0.87 mmol) and **4f** (3.0 h, 196.8 mg, 0.39 mmol, 45%); isomers proportion ~50:50; purple solid; m.p.: 176.5–178.5 °C; IR ν_max_: 2950, 1740, 1710, 1655 and 1626 cm^−1^; ^1^H-NMR: δ 0.87 (t, 6H, *J* = 7.4 Hz CH_3_), 1.53 (s, 3H, CH_3_), 1.67 (s, 3H, CH_3_), 2.36–2.58 (m, 4H, CH_2_), 3.05 (m, 2H, CH_2_), 3.17 (m, 2H, CH_2_), 3.60 (s, 3H, CO_2_CH_3_), 3.62 (s, 3H, CO_2_CH_3_), 3.74 (s, 6H, CO_2_CH_3_), 4.36 (m, 2H, CH), 7.23–7.36 (m, 10H, arom.), 7.75 (m, 4H, arom.), 8.10 (m, 4H, arom.), 9.92 (br s, 2H, NH). ^13^C-NMR: δ 13.7, 13.8, 16.3, 16.5, 17.2, 17.4, 40.2, 40.3, 45.0, 51.1, 52.7, 52.8, 58.2, 58.3, 126.3, 127.0, 127.1, 127.4, 128.9, 129.0, 129.3, 129.5, 132.0, 132.2, 134.0, 134.2, 135.8, 135.9, 141.7, 147.1, 168.0, 168.1, 171.4, 171.5, 184.0, 184.8, 202.9. HRMS (M^+^): *m*/*z* calcd. for C_29_H_29_NO_7_: 503.1944; found: 503.2008.

*Methyl (Z)-[3-(3-hexanoyl-1,4-dioxo-1,4-dihydronaphthalen-2-yl)-4-methoxy-4-oxobut-2-en-2-yl]-l-leucinate* (**13**). Prepared from **1d** (100 mg, 0.38 mmol) and **4d** (2.5 h, 75.88 mg, 0.15 mmol, 40%); isomers proportion ~44:56; violet oil; IR ν_max_: 2995, 1756, 1690, 1678 and 1645 cm^−1^; ^1^H-NMR: δ 0.84 (m, 6H, CH_3_), 0.89–0.95 (m, 12H, CH_3_), 1.24 (m, 8H, CH_2_), 1.58 (m, 4H, CH_2_), 1.61–1.72 (m, 6H, CH and CH_2_), 1.74 (s, 6H, CH_3_), 1.76 (s, 6H, CH_3_), 2.51 (m, 4H, CH_2_), 3.56 (s, 6H, CO_2_CH_3_), 3.73 (s, 6H, CO_2_CH_3_), 4.13 (m, 2H, CH), 7.73 (m, 4H, arom.), 8.04–8.07 (m, 4H, arom.), 9.65 (d, 1H, *J* = 7.2 Hz), 9.76 (d, 1H, *J* = 6.8 Hz). ^13^C-NMR: δ 13.91 17.43, 21.98, 22.01, 22.53 (2C), 22.69, 22.75, 24.71, 24.78, 31.22, 41.97, 42.32, 42.92, 50.95, 52.51, 52.58, 54.90, 54.94, 87.34, 126.21, 126.25, 126.91, 126.97, 131.94, 131.97, 132.12, 132.14, 133.92, 133.96, 134.11, 134.14, 141.61, 141.72, 147.22, 161.38, 168.11, 172.39, 172.54, 183.84, 184.69, 184.99, 2002.74, 202.91. HRMS (M^+^): *m*/*z* calcd. for C_28_H_35_NO_7_: 497.2414; found: 497.2479.

*Methyl (S,Z)-3-[(3-(1H-indol-3-yl)-1-methoxy-1-oxopropan-2-yl)amino]-2-(3-hexanoyl-1,4-dioxo-1,4-dihydronaphthalen-2-yl)but-2-enoate* (**14**). Prepared from **1d** (100 mg, 0.38 mmol) and **4g** (2.5 h, 126.1 mg, 0.22 mmol, 58%); isomers proportion ~45:55; red solid; m.p.: 264.5–266.5 °C; IR ν_max_: 2990, 1735, 1697, 1670 and 1650 cm^−1^; ^1^H-NMR: δ 0.88 (t, 3H, *J* = 7.8 Hz, CH_3_), 0.90 (t, 3H, *J* = 7.8 Hz, CH_3_), 1.20–1.35 (m, 8H, CH_2_), 1.41 (s, 3H, CH_3_), 1.49 (s, 3H, CH_3_), 2.47–2.65 (m, 4H, CH_2_), 3.13–3.25 (m, 2H, CH_2_), 3.36 (m, 1H, CH_2_), 3.40 (m, 1H, CH_2_), 3.60 (s, 6H, CO_2_CH_3_), 3.72 (s, 6H, CO_2_CH_3_), 4.41 (ddd, 2H, *J* = 5.0, 8.1 and 16.7 Hz, CH), 7.0 (s, 1H, CH), 7.09–7.19 (m, 5H, arom.), 7.32 (d, 1H, *J* = 7.6 Hz, arom.), 7.37 (d, 1H, *J* = 8.0 Hz, arom.), 7.57 (d, 1H, *J* = 7.6 Hz, arom.), 7.76 (m, 4H, arom.), 8.06–8.14 (m, 4H, arom.), 8.33 (br s, 1H, NH), 8.44 (br s, 1H, NH), 9.95 (d, 1H, *J* = 8.4 Hz, NH), 10.12 (br s, 1H, NH). ^13^C-NMR: δ 14.0, 14.1, 17.1, 17.2, 22.5, 22.55, 22.57, 22.76, 30.1, 30.5, 31.3, 31.3, 43.0, 43.1, 50.9, 51.0, 52.7, 52.8, 57.0, 57.2, 109.2, 109.6, 111.5, 111.6, 118.2, 118.3, 119.7, 122.2, 122.3, 123.9, 124.5, 126.2, 126.3, 126.9, 127.1, 127.1, 131.9, 132.0, 132.1, 132.3, 133.9, 134.0, 134.15, 134.20, 136.2, 136.3, 141.8, 142.0, 147.21, 161.2, 168.0, 169.0, 171.7, 171.8, 183.9, 184.1, 184.8, 185.1, 202.9. HRMS (M^+^): *m*/*z* calcd. for C_33_H_34_N_2_O_7_: 570.2366; found: 570.2434.

### 3.4. Cytotoxicity Assay 

Cell Lines and Culture Conditions: MRC-5 normal human lung fibroblasts (CCL-171), AGS human gastric adenocarcinoma cells (CRL-1739), SK-MES-1 human lung cancer cells (HTB-58), and J82 human bladder carcinoma cells (HTB-1) were obtained from the American Type Culture Collection (ATCC, Manassas, VA, USA). MRC-5, SK-MES-1, and J82 cells were grown in Eagle’s minimal essential medium (EMEM) containing 2 mM l-glutamine, 1 mM sodium pyruvate and 1.5 g/L sodium hydrogen carbonate. AGS cells were grown in Ham F-12 supplemented with 2 mM l-glutamine and 1.5 g/L sodium hydrogen carbonate. Finally, HL-60 cells were grown in RPMI medium. Media were supplemented with 10% heat-inactivated fetal bovine serum (FBS), 100 IU/mL penicillin and 100 μg/mL streptomycin and cell cultures were kept in a humidified incubator with 5% CO2 in air at 37 °C. For the cytotoxicity experiments, cells were seeded into 96-well plates at a density of 50,000 cells/mL. After reaching confluence, cells were incubated for three days with compounds at varied concentrations ranging from 0 up to 100 μM while untreated cells (medium containing 1% DMSO) were used as controls. Cytotoxicity was assessed using the MTT (3-(4,5-dimethylthiazol-2-yl)-2,5-diphenyltetrazolium bromide) reduction assay. MTT was used at 1 mg/mL and the blue formazan crystals, formed during MTT reduction, were dissolved adding 100 μL of ethanol (acidified with HCl). The absorbance was measured at 550 nm using a Universal Microplate Reader (ELX 800, Bio-Tek Instruments Inc., Winooski, VT, USA). Values were the means of six replicates for each concentration and transformed to percentage of controls. The IC_50_ value was graphically obtained from the dose–response curves by adjusting them to a sigmoidal model (a + (b − a)/1 + 10(*x* − c)), where c = log IC_50_.

## 4. Conclusions

In summary, we have studied the reactivity of a number of 2-acylnaphthoquinones with secondary enaminones derived from 2-aminoethanol and α-amino acid methyl esters. The reactions provided access to 1,2-dihydrobenzisoquinolinequinones **5a** and **5b** and a variety of napthoquinone-amino ester conjugates **6**–**14**. The main novelty of the study concerns the facile access, and with high atom economy, to a new scaffold containing the biological relevant naphthoquinone and amino acid fragments, bonded through a vinyl spacer. The preliminary results on the biological evaluation of conjugates **6**–**14** showed interesting in vitro cytotoxic activity on cancer cells.

## Figures and Tables

**Table 1:**
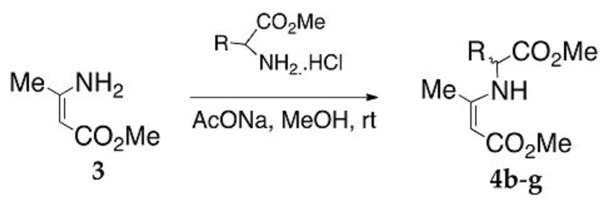
Structure and yields of enaminones **4b**–**g**.

Compound No.	Structure	Yield (%) *	Compound No.	Structure	Yield (%) *
**4b**	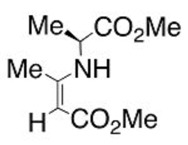	71	**4e**	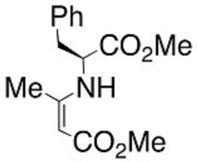	83
**4c**	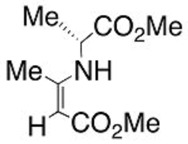	85	**4f**	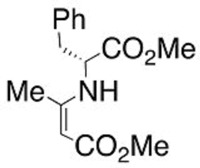	76
**4d**	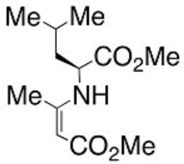	80	**4g**	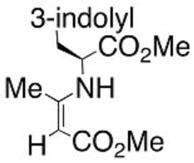	84

* Isolated by column chromatography.

**Table 2:**
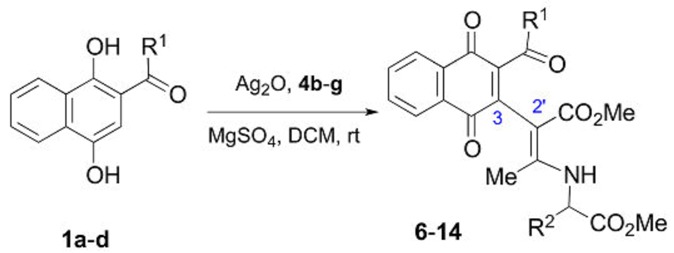
Yields of acylnaphthoquinone α-aminoesters conjugate **6**–**14**.

Amino Acid	R^1^	R^2^	Products	Yield * (%)
l-Ala	Ph	CH_3_	**6**	58
d-Ala	Ph	CH_3_	**7**	52
l-Leu	Ph	(CH_3_)_2_CHCH_2_	**8**	42
l-Phe	Ph	PhCH_2_	**9**	65
d-Phe	Ph	PhCH_2_	**10**	58
l-Trp	Ph	3-IndolylCH_2_	**11**	71
d-Phe	C_3_H_7_	PhCH_2_	**12**	45
l-Leu	C_5_H_11_	(CH_3_)_2_CHCH_2_	**13**	40
l-Trp	C_5_H_11_	3-IndolylCH_2_	**14**	58

* Isolated by column chromatography.

**Table 3:** IC_50_ values of acylnaphthoquinone α-aminoesters conjugates **6**–**14**. IC_50_ ± SEM (μM) ^a^.

Product Number	MRC-5 ^b^	AGS ^c^	SK-MES-1 ^d^	J82 ^e^
**6**	58.0 ± 4.1	52.7 ± 3.7	47.8 ± 3.3	37.1 ± 2.2
**7**	17.4 ± 0.7	20.5 ± 1.1	53.4 ± 4.3	35.6 ± 1.4
**8**	26.6 ± 1.3	26.9 ± 1.5	34.5 ± 1.7	12.9 ± 0.9
**9**	33.3 ± 2.2	19.3 ± 1.5	39.3 ± 1.6	22.0 ± 1.1
**10**	46.7 ± 2.9	30.7 ± 1.5	34.2 ± 2.3	23.0 ± 0.7
**11**	13.0 ± 0.8	10.1 ± 0.1	18.9 ± 0.5	21.4 ± 0.8
**12**	21.8 ± 1.5	12.8 ± 0.7	15.9 ± 0.9	14.0 ± 0.6
**13**	24.9 ± 0.7	17.0 ± 1.1	21.6 ± 1.3	23.5 ± 1.6
**14**	22.1 ± 1.6	9.3 ± 0.6	5.5 ± 0.2	4.5 ± 0.2
Ref. ^f^	2.2 ± 0.1	0.4 ± 0.0	2.9 ± 0.2	3.3 ± 0.2

^a^ Data represent average values of six independent determinations; ^b^ Normal human lung fibroblasts cells; ^c^ Human gastric adenocarcinoma cell line; ^d^ Human lung cancer cell line; ^e^ Human bladder carcinoma cell line; ^f^ Ref.: etoposide

## Supplementary Materials

Supplementary File 1
