# Supplementary material for: Access to New Cytotoxic Quinone-Amino Acid Conjugates Linked through A Vinylic Spacer from 2-Acylnaphthoquinones and Methyl 3-Aminocrotonate
Source: Molecules. 2017 Dec 20;22(12):2281. doi: 10.3390/molecules22122281 (PMC6149812; doi:10.3390/molecules22122281)
Supplement: Supplementary File 1 [file molecules-22-02281-s001.pdf]

Molecules Journal

Supplementary Information

Access to new cytotoxic quinone-amino acid conjugates linked through a vinylic spacer from 2-acylnaphthoquinones and methyl 3-aminocrotonate

Jaime A. Valderrama[1,2*], Joel Garrido[2], Virginia Delgado[3], Julio Benites[1,2*], and Cristina Theoduloz[4]

[1]*Instituto de Ciencias Exactas y Naturales (ICEN), Universidad Arturo Prat Casilla 121, Iquique 1100000, Chile; jaimeadolfov@gmail.com; juliob@unap.cl*

[2]*Química y Farmacia. Facultad de Ciencias de la Salud, Universidad Arturo Prat, Casilla 121, Iquique 1100000, Chile; jaimeadolfov@gmail.com; juliob@unap.cl*

[3]*Facultad de Química, Pontificia Universidad Católica de Chile, Casilla 306, Santiago 6094411, Chile; vcdelgad@uc.cl*

[4]*Facultad de Ciencias de la Salud, Universidad de Talca, Talca 3460000, Chile; ctheodul@utalca.cl*

E-mail addresses: jaimeadolfov@gmail.com (J.A. Valderrama); juliob@unap.cl (J. Benites)

Contents

^{1}H NMR spectra coalescence experiments in DMSO-d_6 at temperatures over 25.6 ºC, for compound **7**........ 3

^{1}H NMR spectra coalescence experiments in DMSO-d6 at temperatures over 25.6 ºC, for compound **9** 6

^{1}H, ^{13}C and HMBC NMR spectra of compound **4c** 11

^{1}H, ^{13}C and H-H COSY NMR spectra of compound **4d** 13

^{1}H, ^{13}C, H-H COSY and HMBC NMR spectra of compound **5b** 15

^{1}H, ^{13}C, HMBC and H-H COSY NMR spectra of compound **6** 17

^{1}H, ^{13}C NMR and HMBC spectra of compound **9** 19

Compound 7 (NB-DALA)

26.9ºC

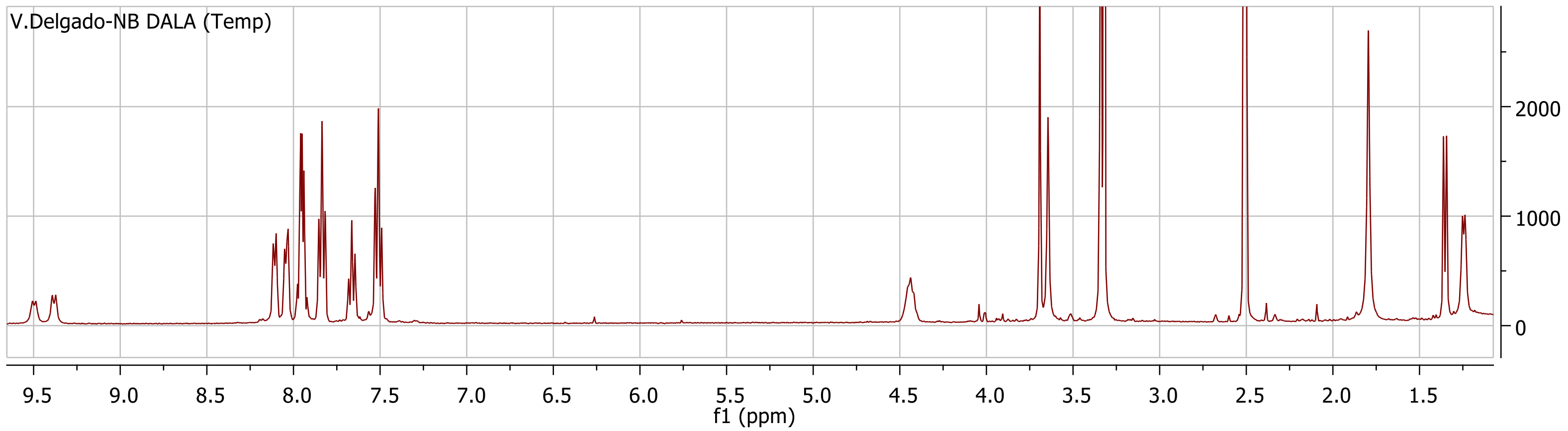

35ºC

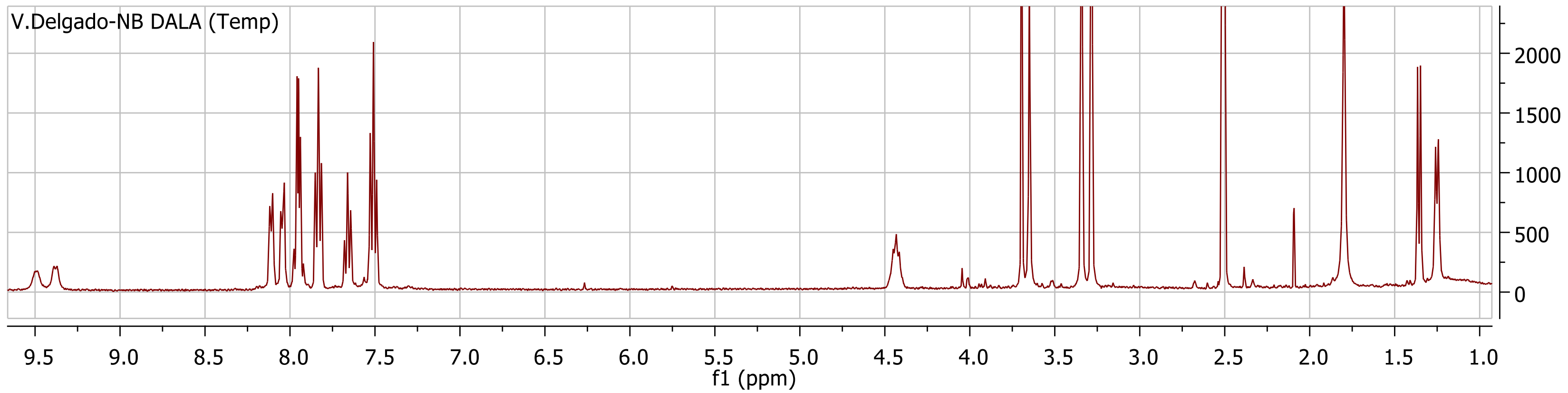

45ºC

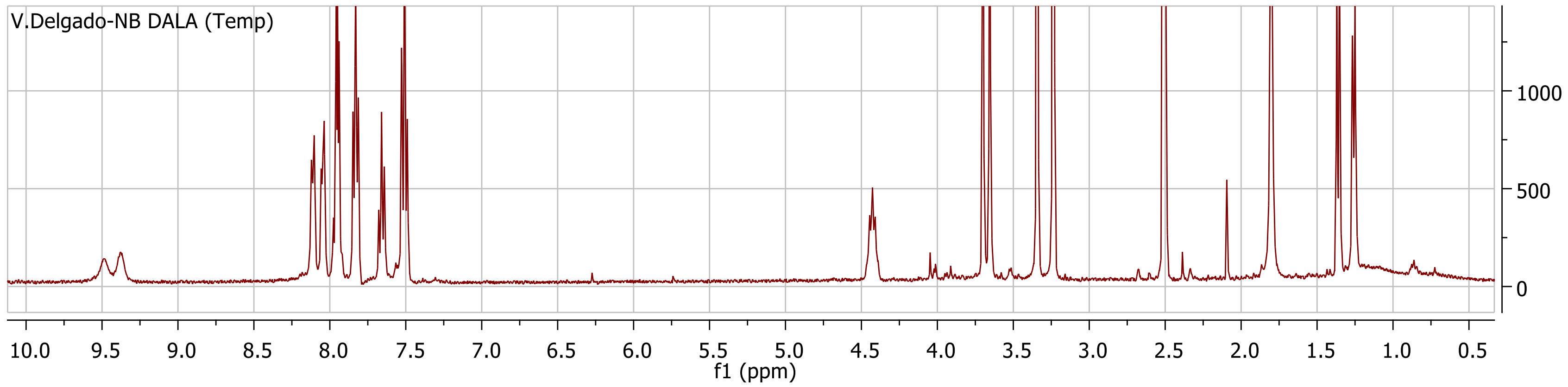

55ºC

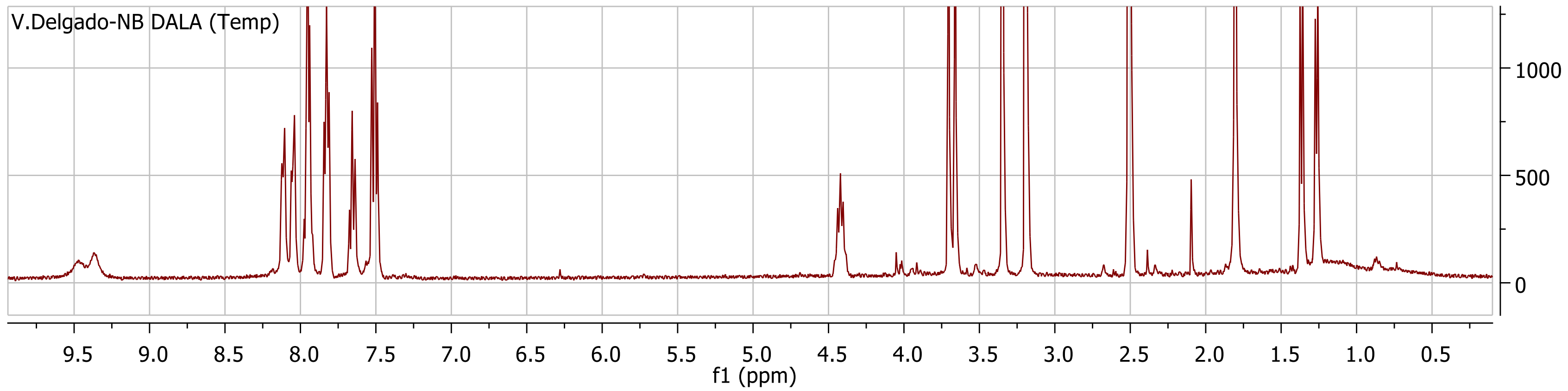

65ºC

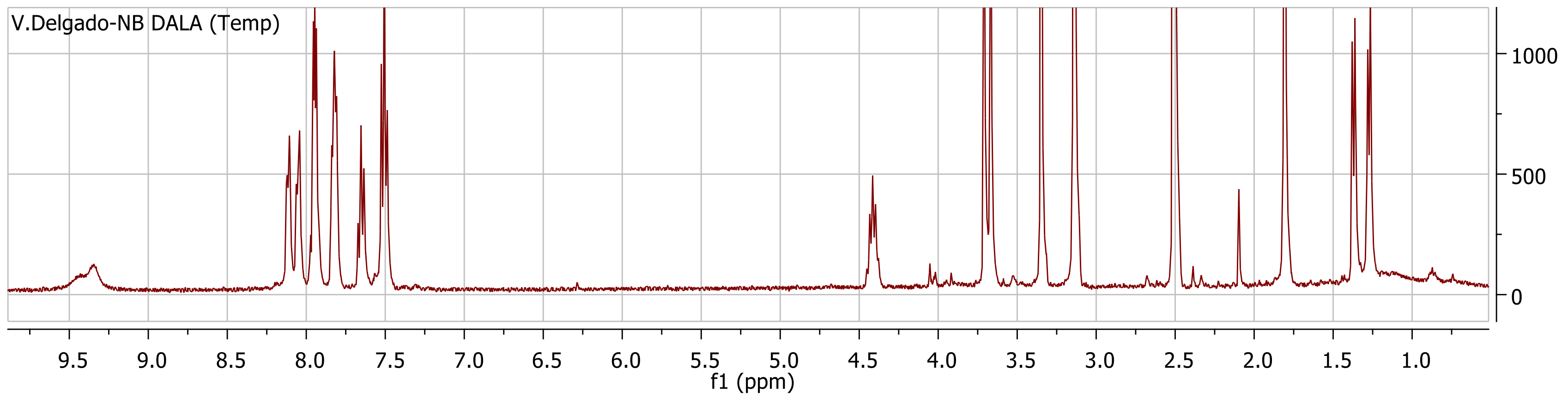
V.Delgado-NB DALA (Temp)
1000
500
0
9.5 9.0 8.5 8.0 7.5 7.0 6.5 6.0 5.5 5.0 4.5 4.0 3.5 3.0 2.5 2.0 1.5 1.0
f1 (ppm)

75ºC

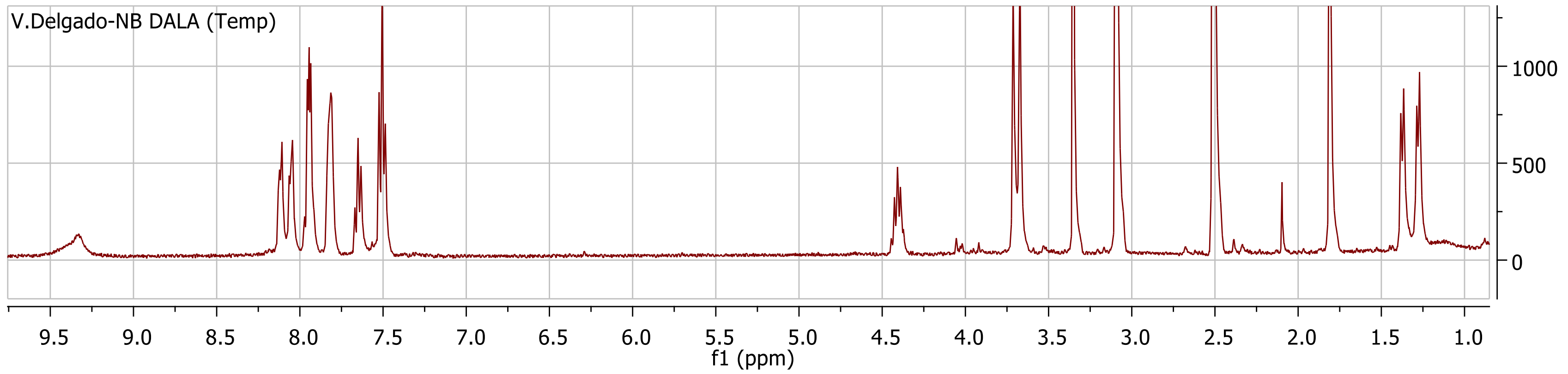
V.Delgado-NB DALA (Temp)
1000
500
0
9.5 9.0 8.5 8.0 7.5 7.0 6.5 6.0 5.5 5.0 4.5 4.0 3.5 3.0 2.5 2.0 1.5 1.0
f1 (ppm)

Compound 9 (NB-LPhe)

26.9 °C

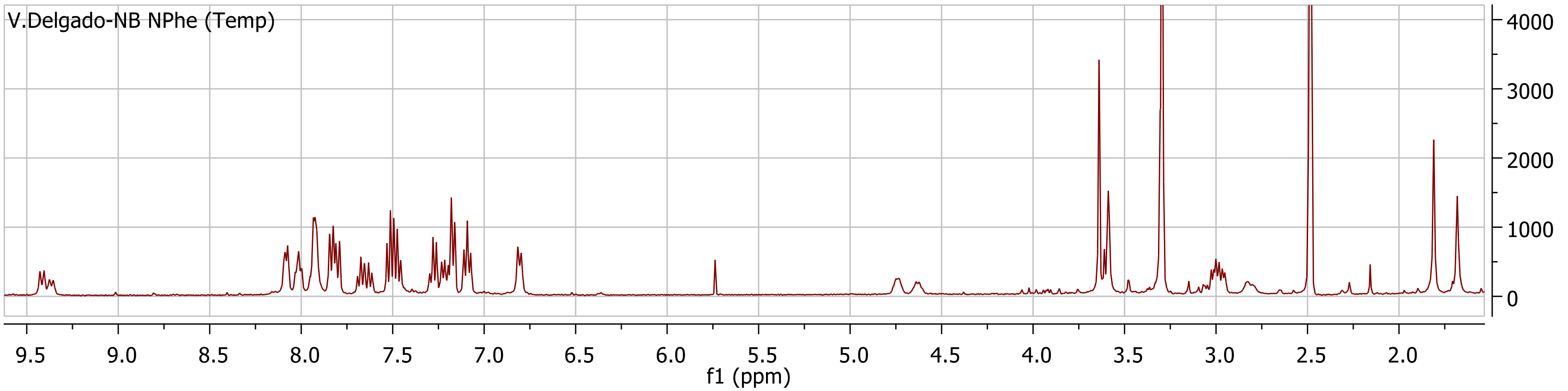

35ºC

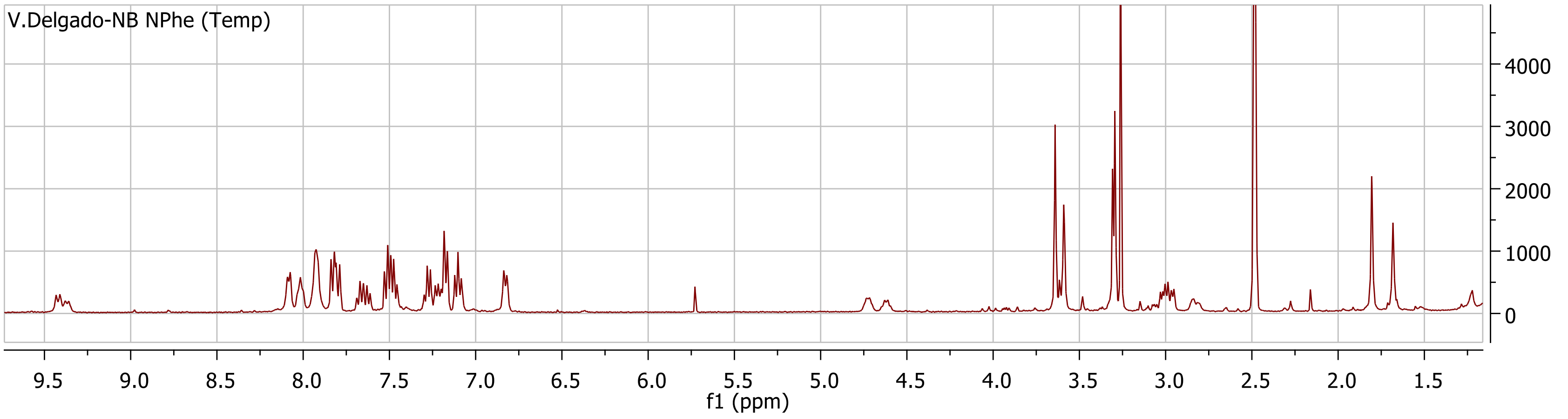

45ºC

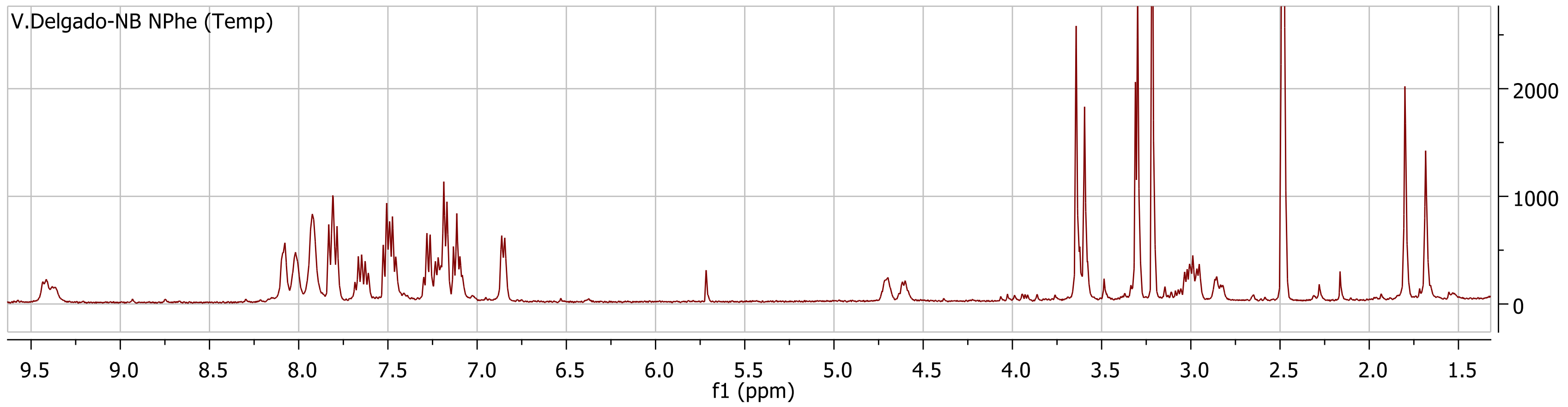

55ºC

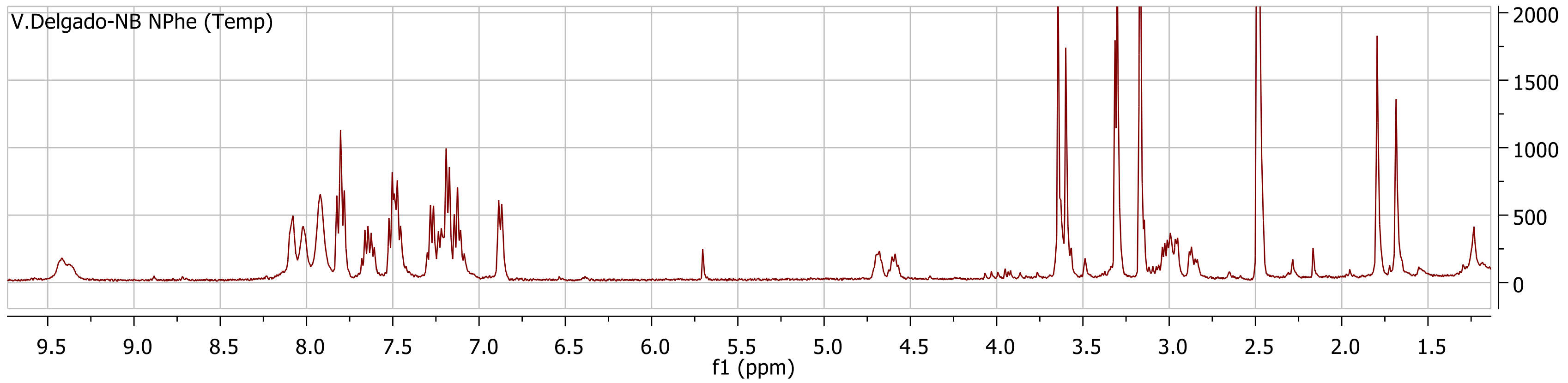

65ºC

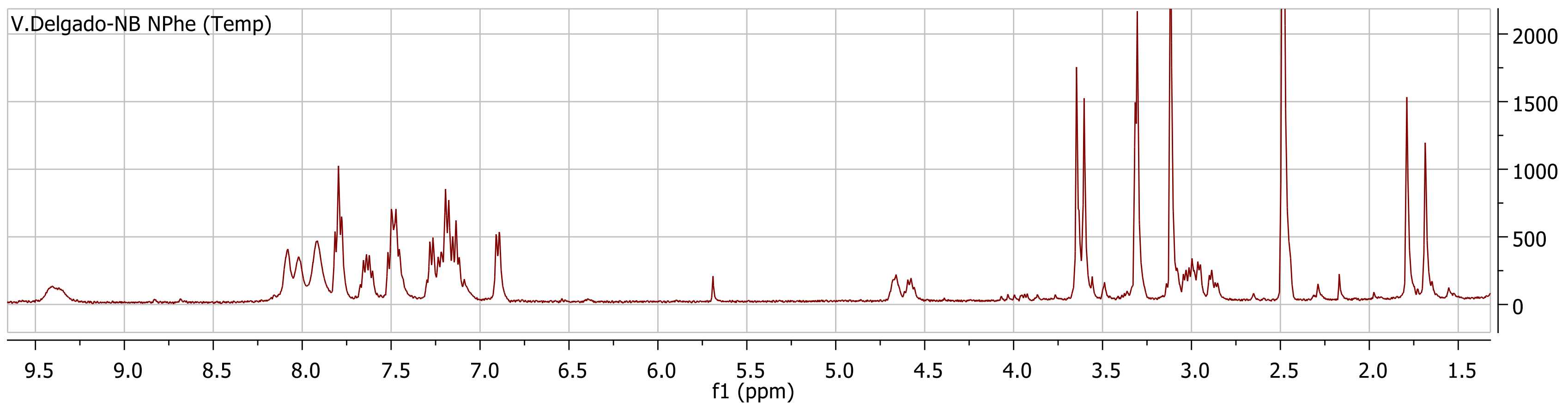

75ºC

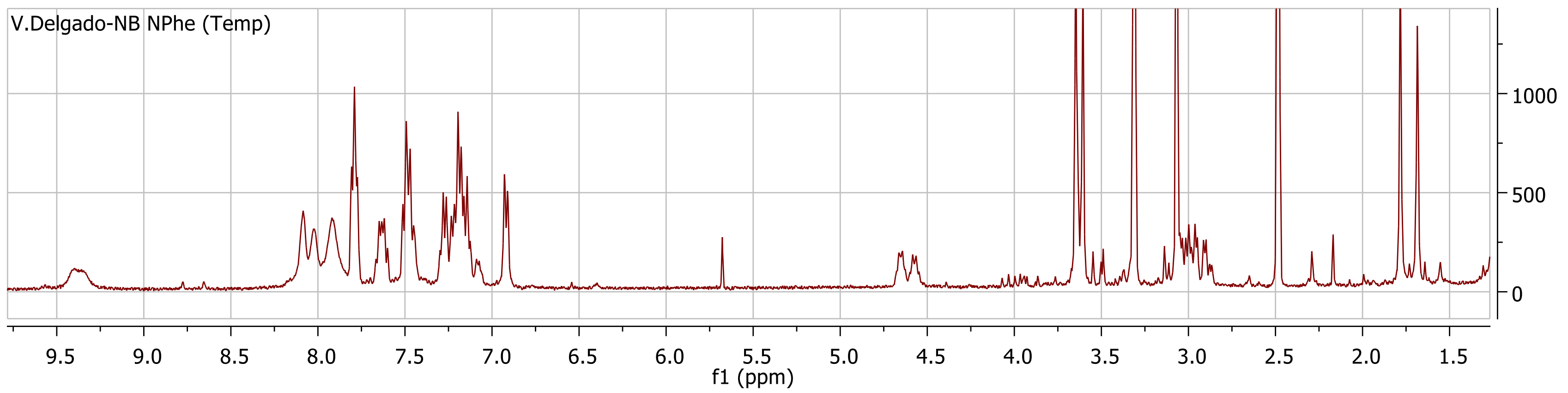

85ºC

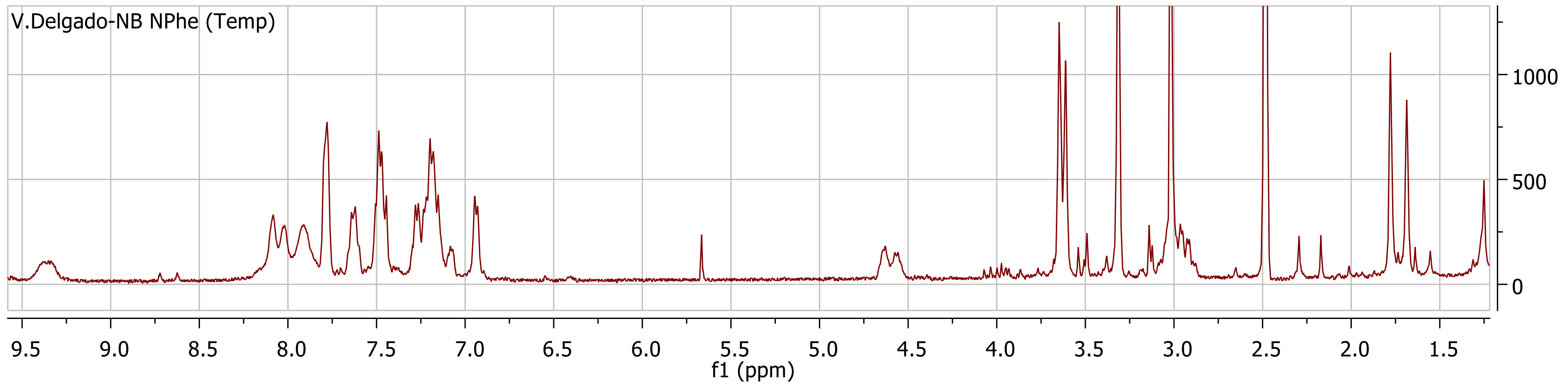

95ºC

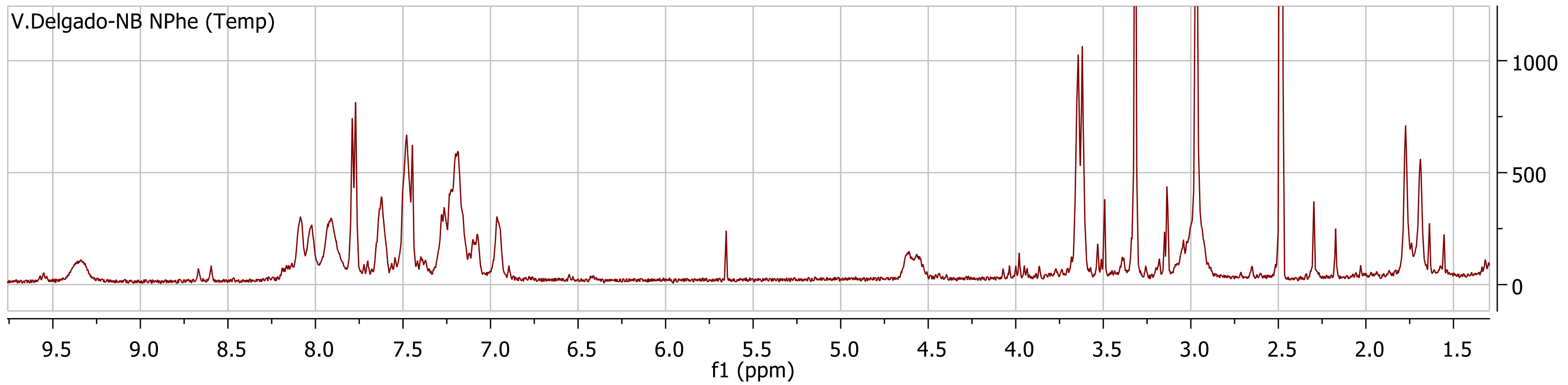

105°C

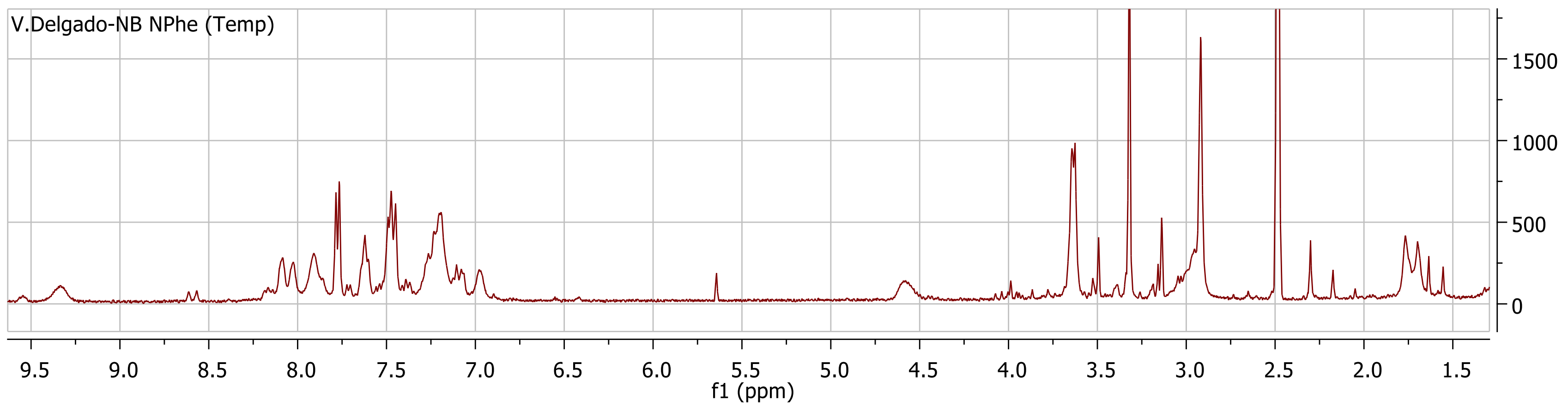

V.Delgado-NB NPhe (Temp)
1500
1000
500
0
9.5
9.0
8.5
8.0
7.5
7.0
6.5
6.0
5.5
5.0
4.5
4.0
3.5
3.0
2.5
2.0
1.5
f1 (ppm)

Compound 4c

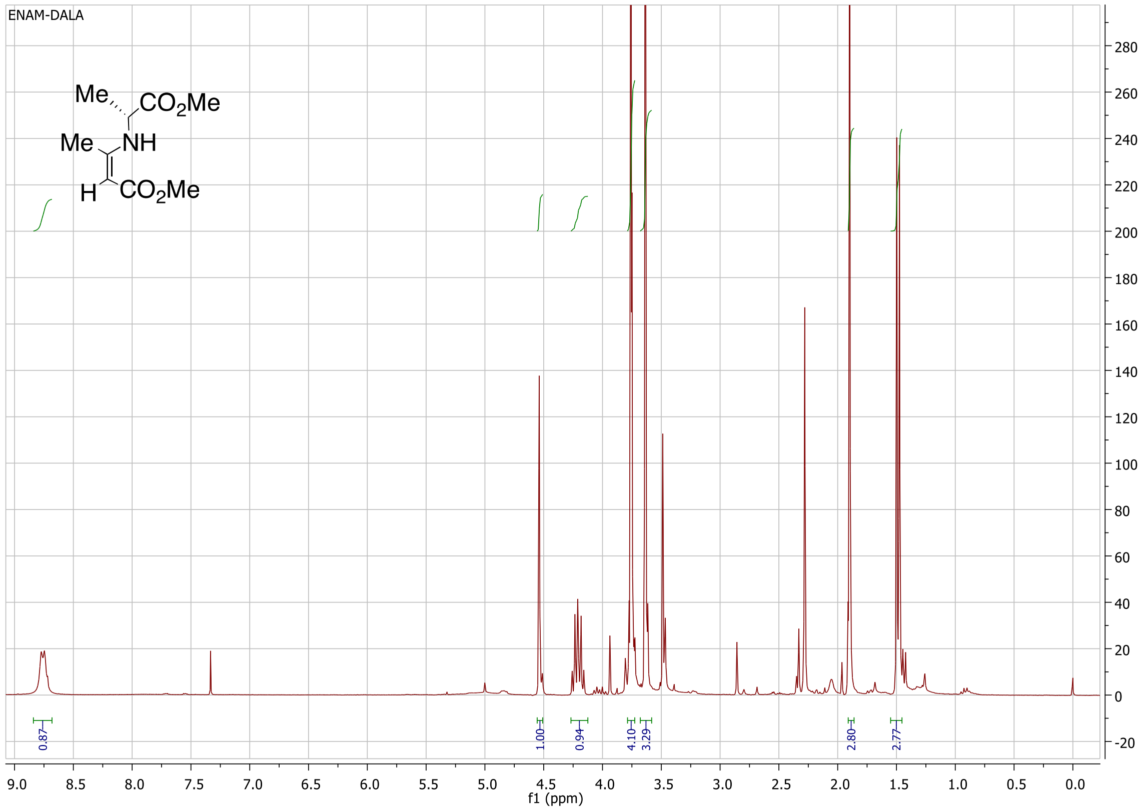

ENAM-DALA
Me
CO2Me
Me
NH
H
CO2Me
0.87
1.00
0.94
4.10
3.29
2.80
2.77
f1 (ppm)

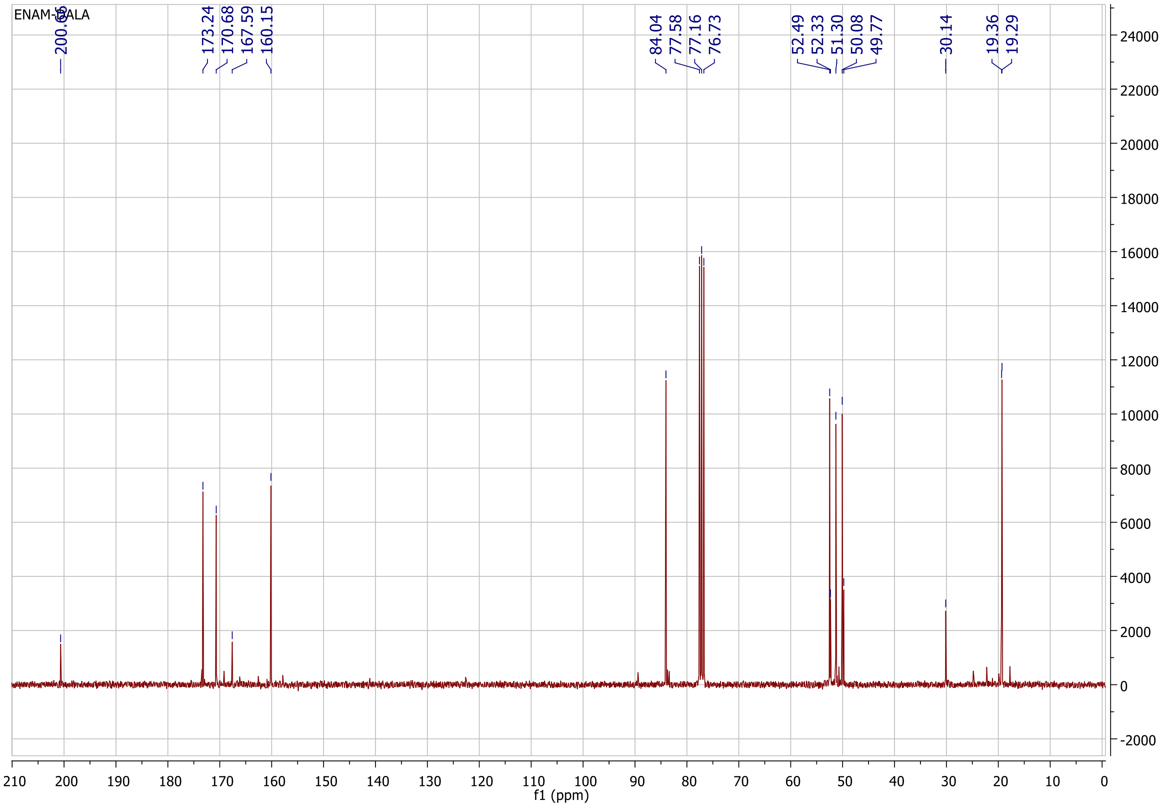

ENAM-DALA
200.66
173.24
170.68
167.59
160.15
84.04
77.58
77.16
76.73
52.49
52.33
51.30
50.08
49.77
30.14
19.36
19.29
f1 (ppm)

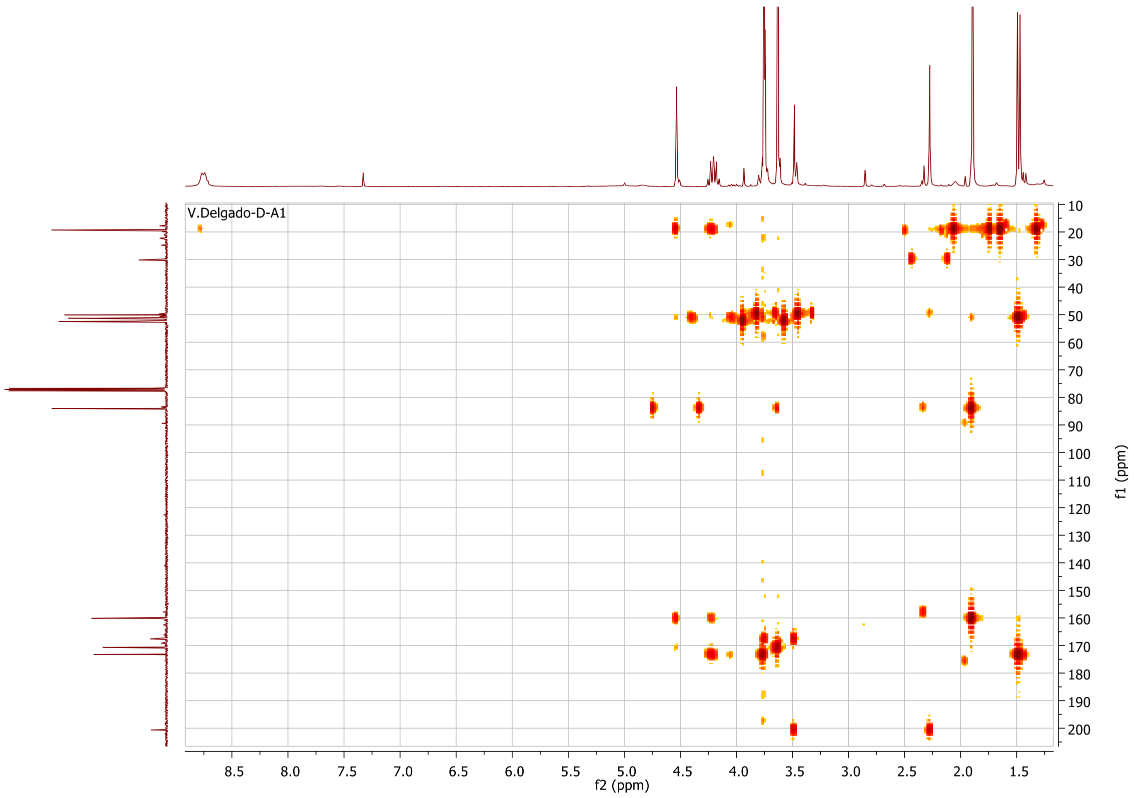
V.Delgado-D-A1
f2 (ppm)
f1 (ppm)

Compound 4d

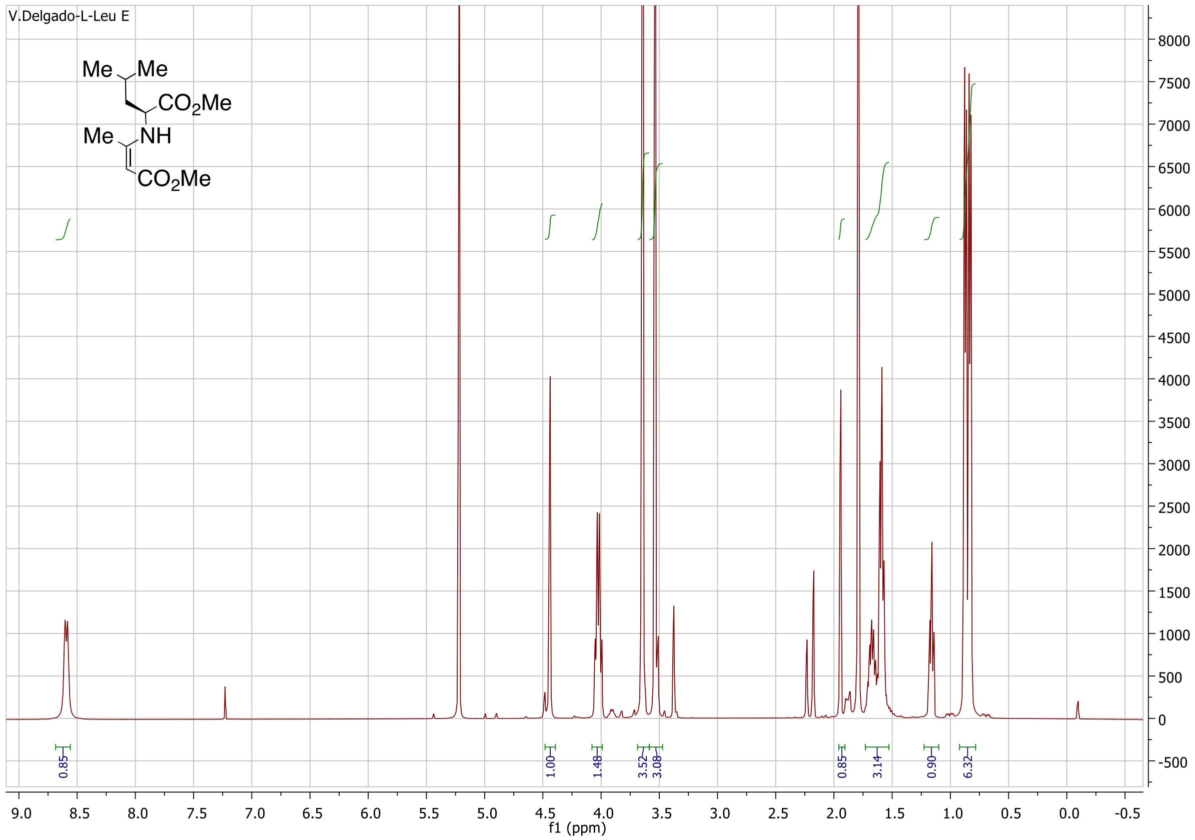
V.Delgado-L-Leu E
Me
Me
CO2Me
Me
NH
CO2Me
0.85
1.00
1.48
3.52
3.08
0.85
3.14
0.90
6.32
f1 (ppm)

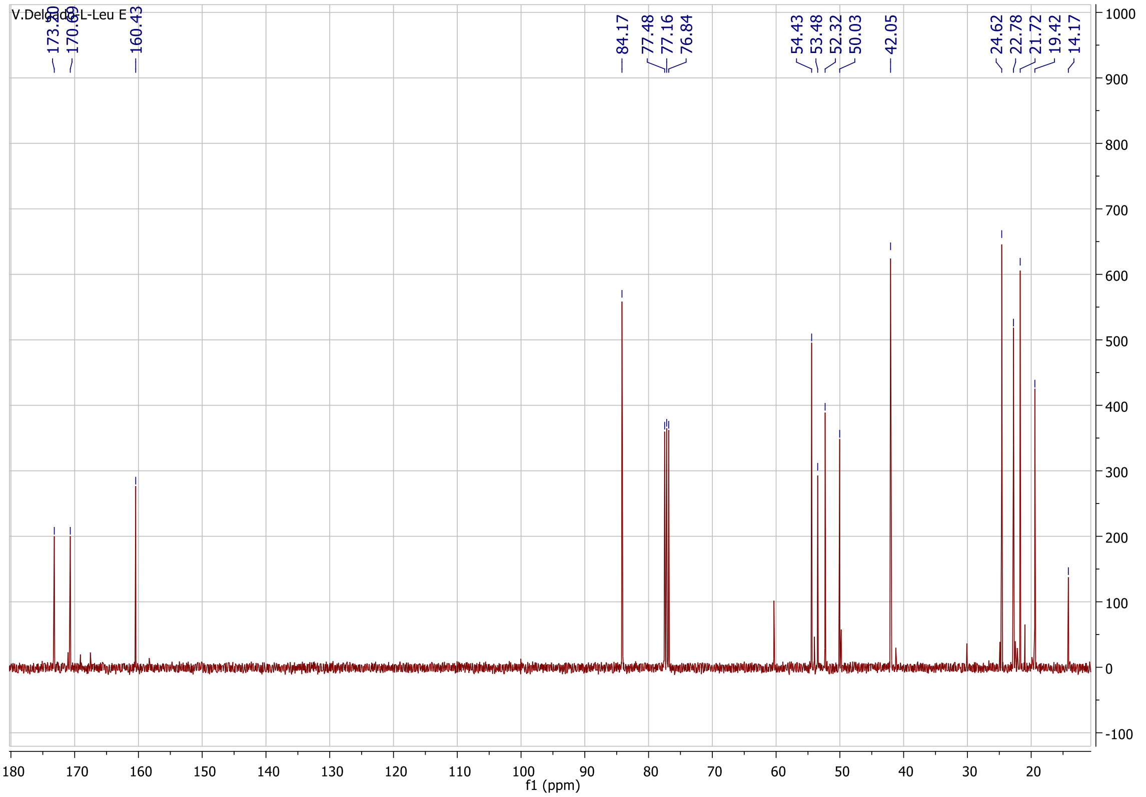
V.Delgado-L-Leu E
160.43
84.17
77.48
77.16
76.84
54.43
53.48
52.32
50.03
42.05
24.62
22.78
21.72
19.42
14.17
f1 (ppm)

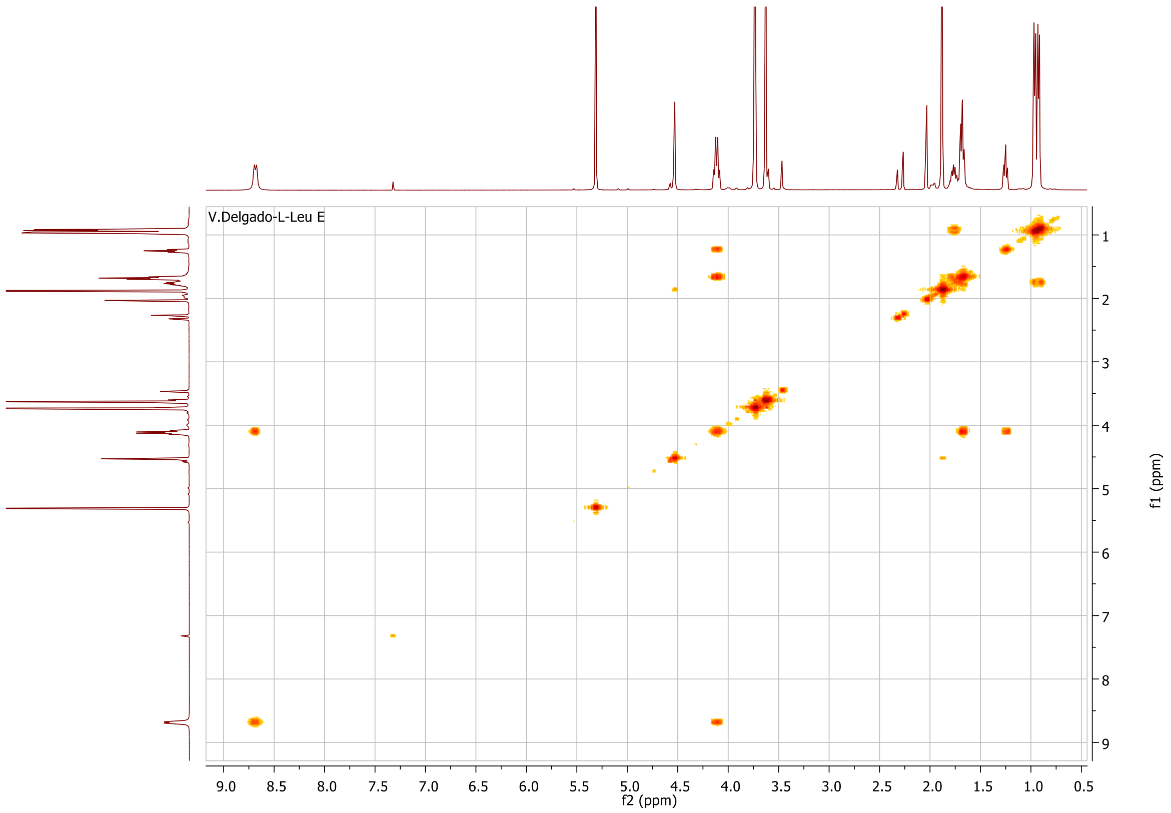
V.Delgado-L-Leu E
9.0 8.5 8.0 7.5 7.0 6.5 6.0 5.5 5.0 4.5 4.0 3.5 3.0 2.5 2.0 1.5 1.0 0.5
f2 (ppm)
1 2 3 4 5 6 7 8 9
f1 (ppm)

Compound 5b

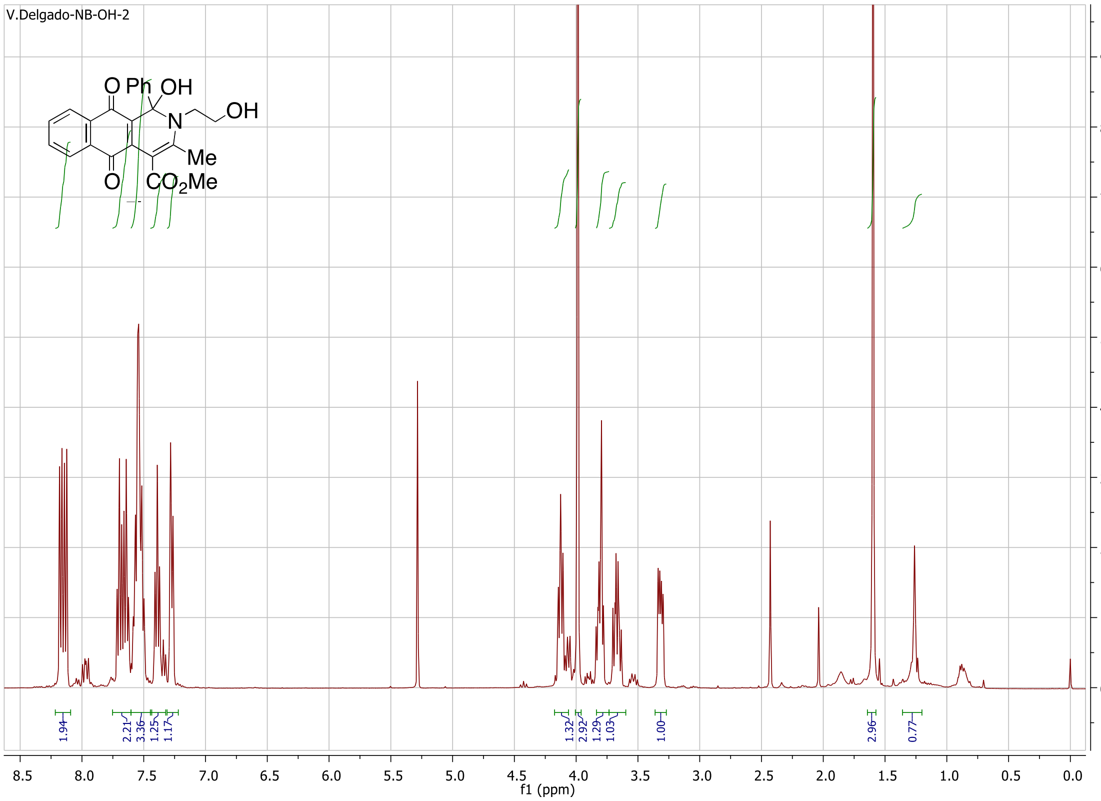
V.Delgado-NB-OH-2
O Ph OH
N OH
Me
O CO2Me
1.94
2.21
3.36
1.25
1.17
1.32
2.92
1.29
1.03
1.00
2.96
0.77
f1 (ppm)

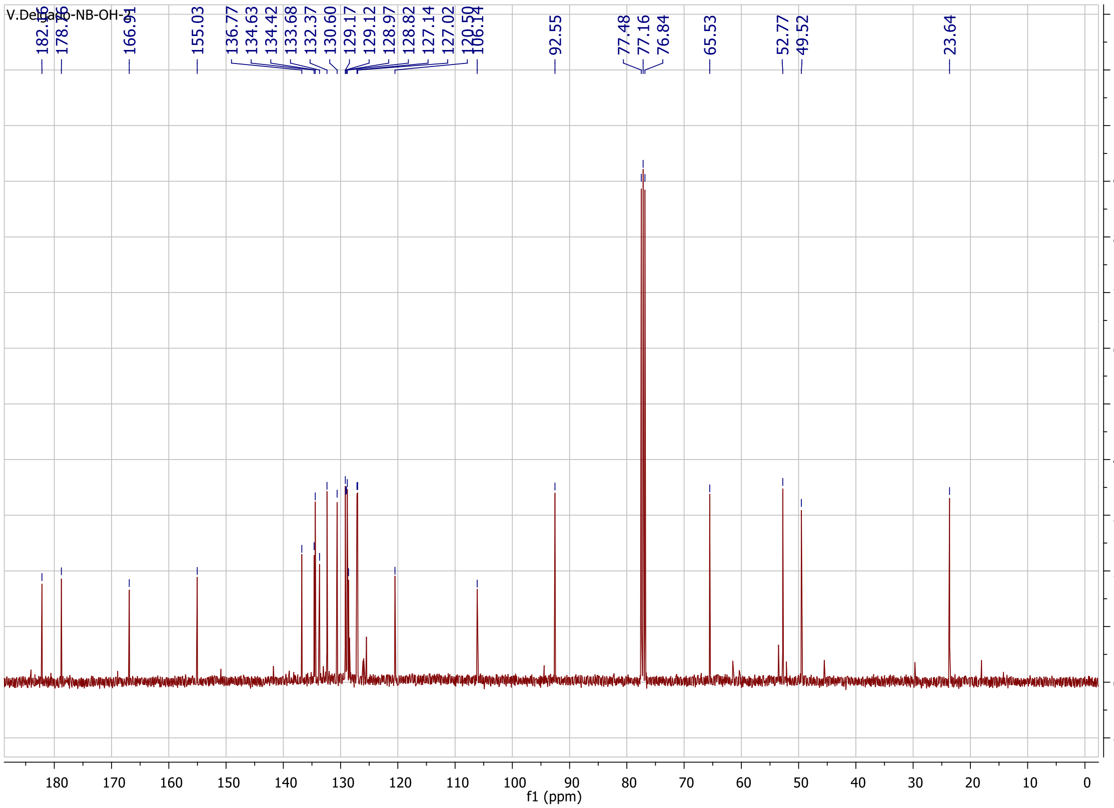
182.16
178.86
166.91
155.03
136.77
134.63
134.42
133.68
132.37
130.60
129.17
129.12
128.97
128.82
127.14
127.02
120.50
106.14
92.55
77.48
77.16
76.84
65.53
52.77
49.52
23.64
f1 (ppm)

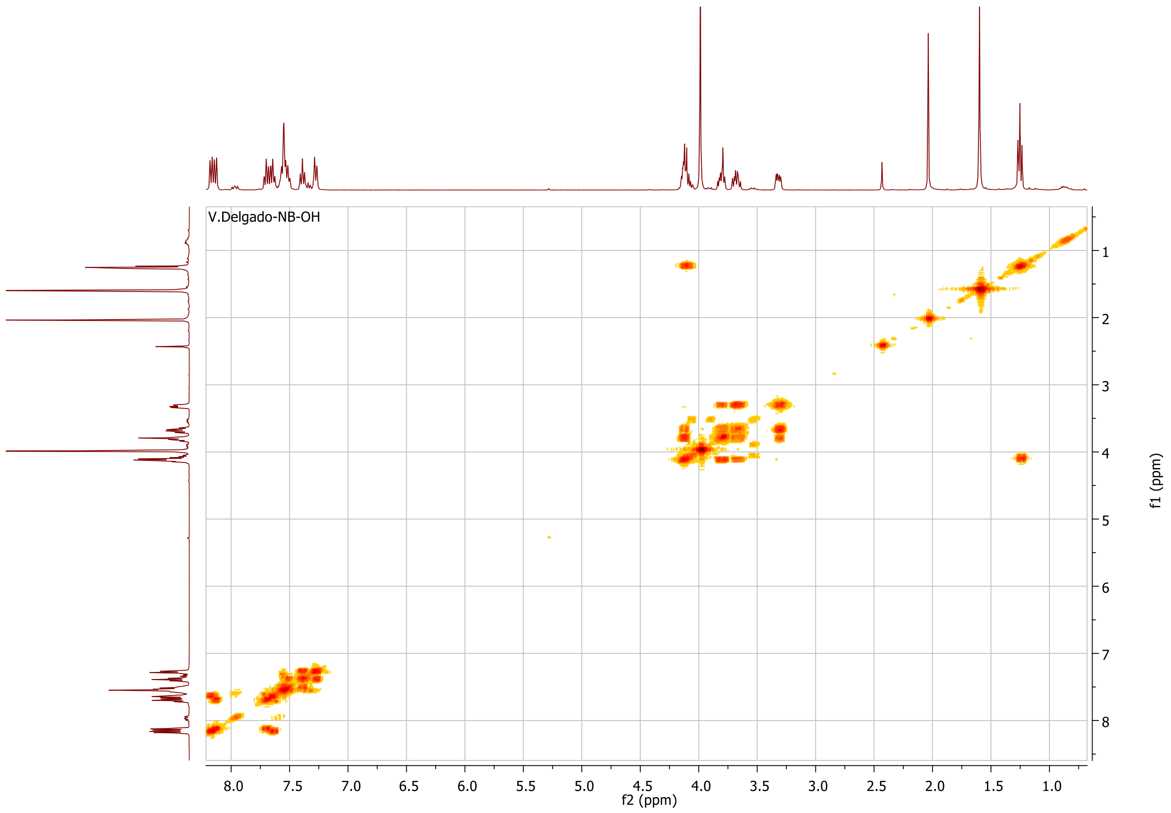
V.Delgado-NB-OH
f2 (ppm)
f1 (ppm)

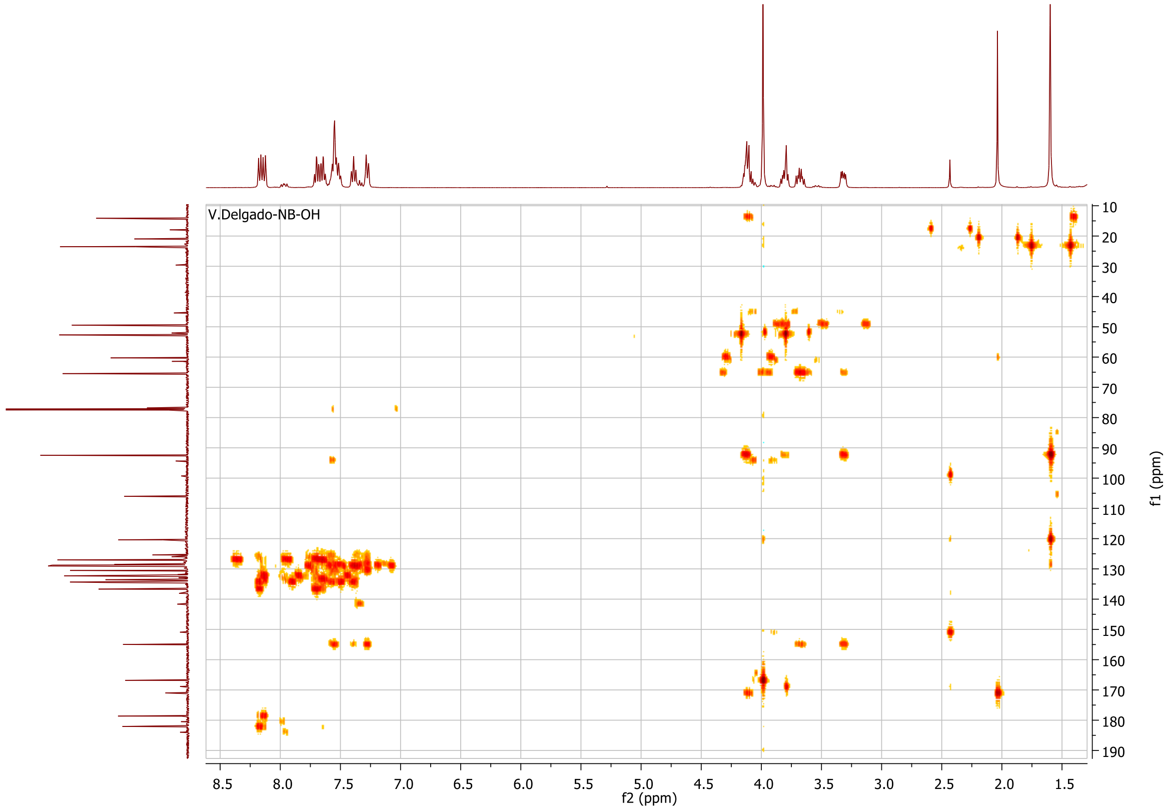
V.Delgado-NB-OH
f2 (ppm)
f1 (ppm)

Compound 6

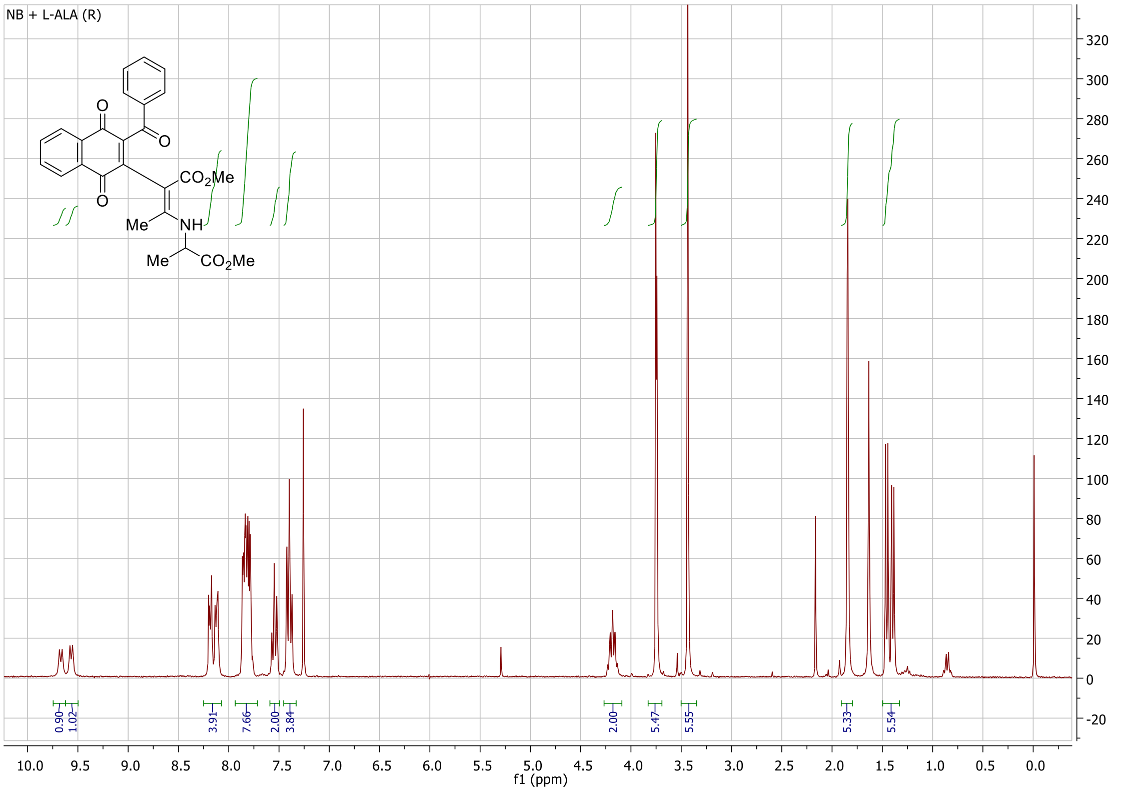
NB + L-ALA (R)
O
O
O
CO$_2$Me
Me
NH
Me
CO$_2$Me
0.90
1.02
3.91
7.66
2.00
3.84
2.00
5.47
5.55
5.33
5.54
f1 (ppm)

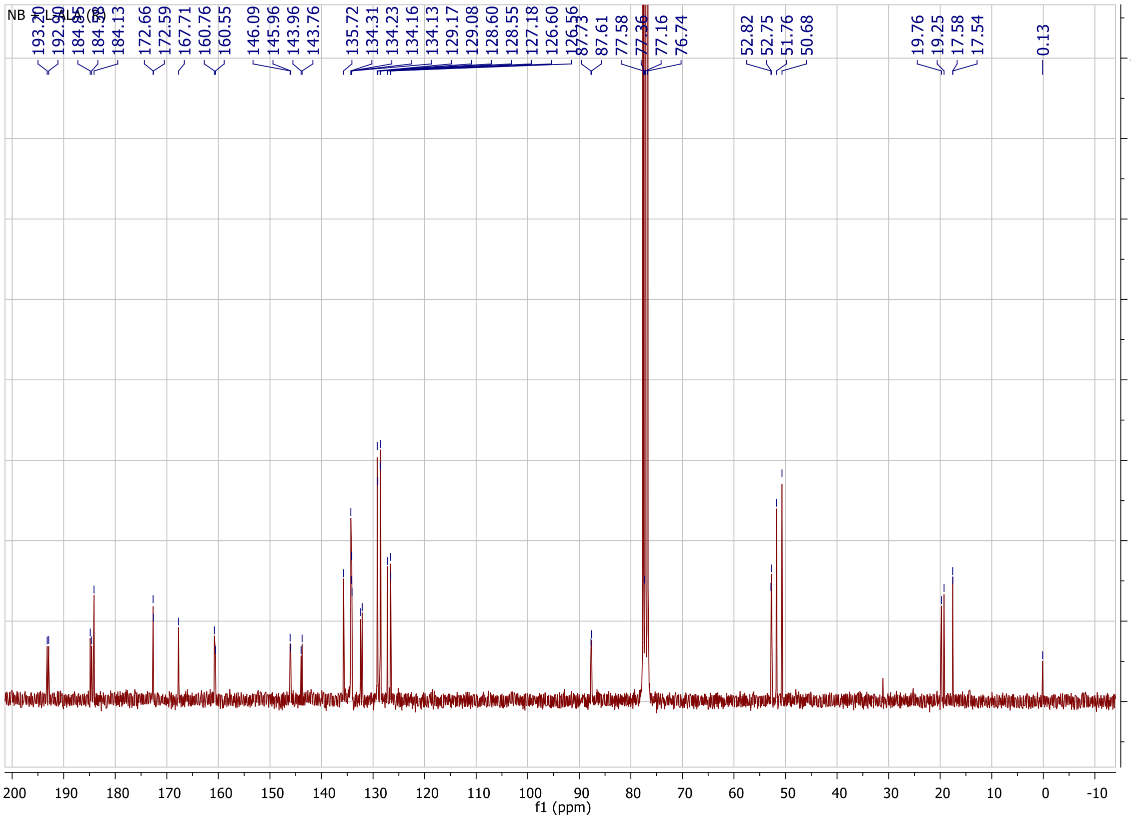
184.13
172.66
172.59
167.71
160.76
160.55
146.09
145.96
143.96
143.76
135.72
134.31
134.23
134.16
134.13
129.17
129.08
128.60
128.55
127.18
126.60
87.73
87.61
77.58
77.16
76.74
52.82
52.75
51.76
50.68
19.76
19.25
17.58
17.54
0.13
f1 (ppm)

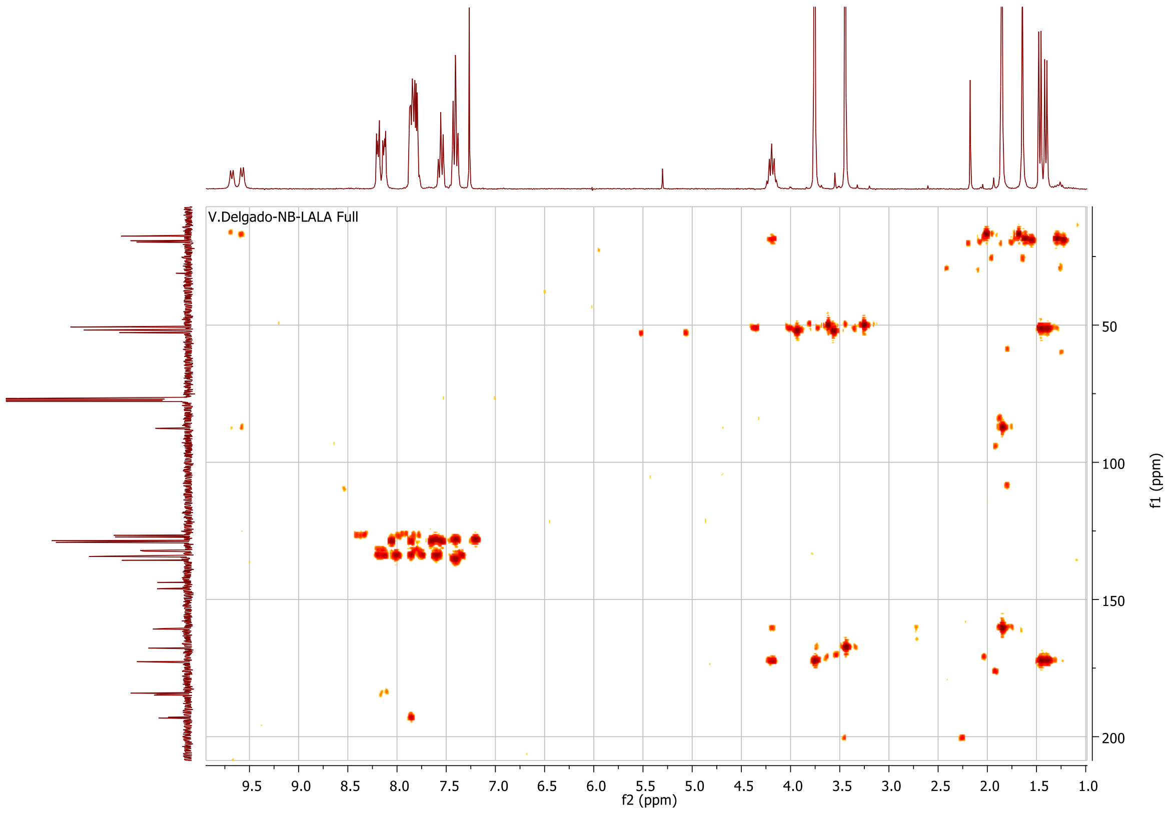
V.Delgado-NB-LALA Full
f2 (ppm)
f1 (ppm)

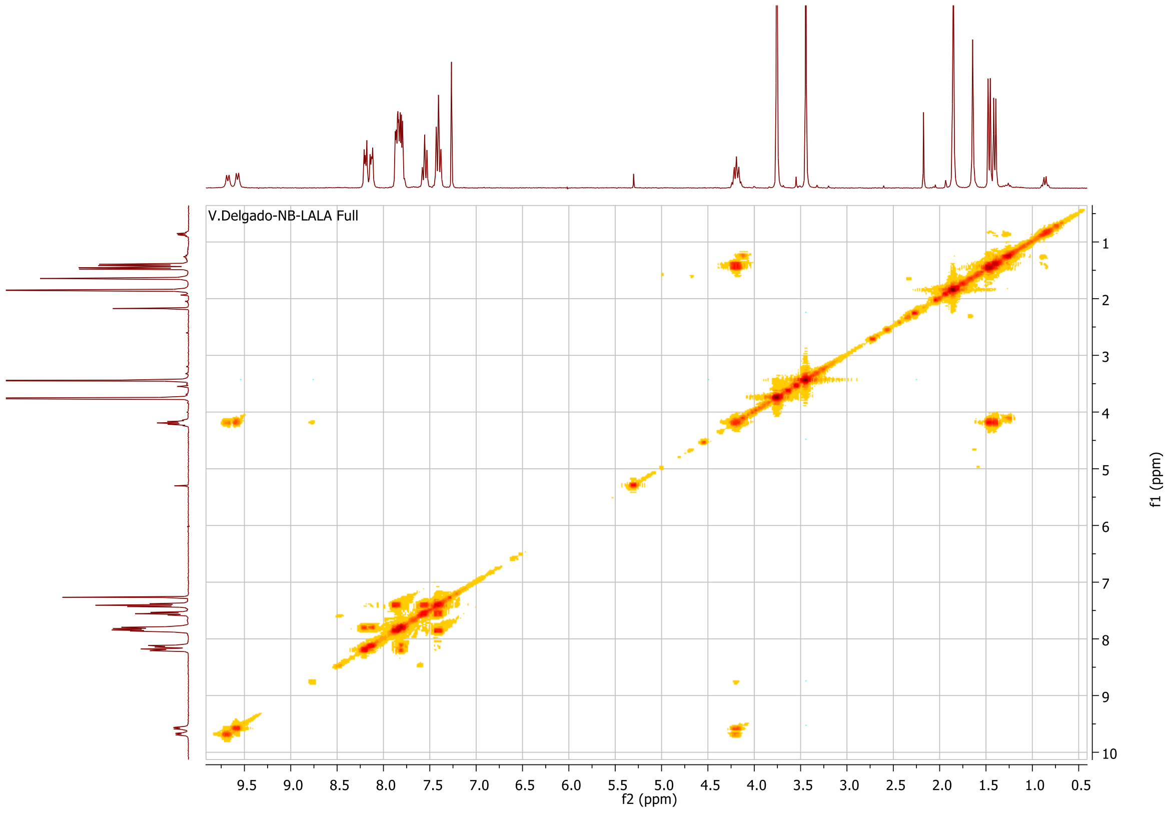
V.Delgado-NB-LALA Full
f2 (ppm)
f1 (ppm)

Compound 9

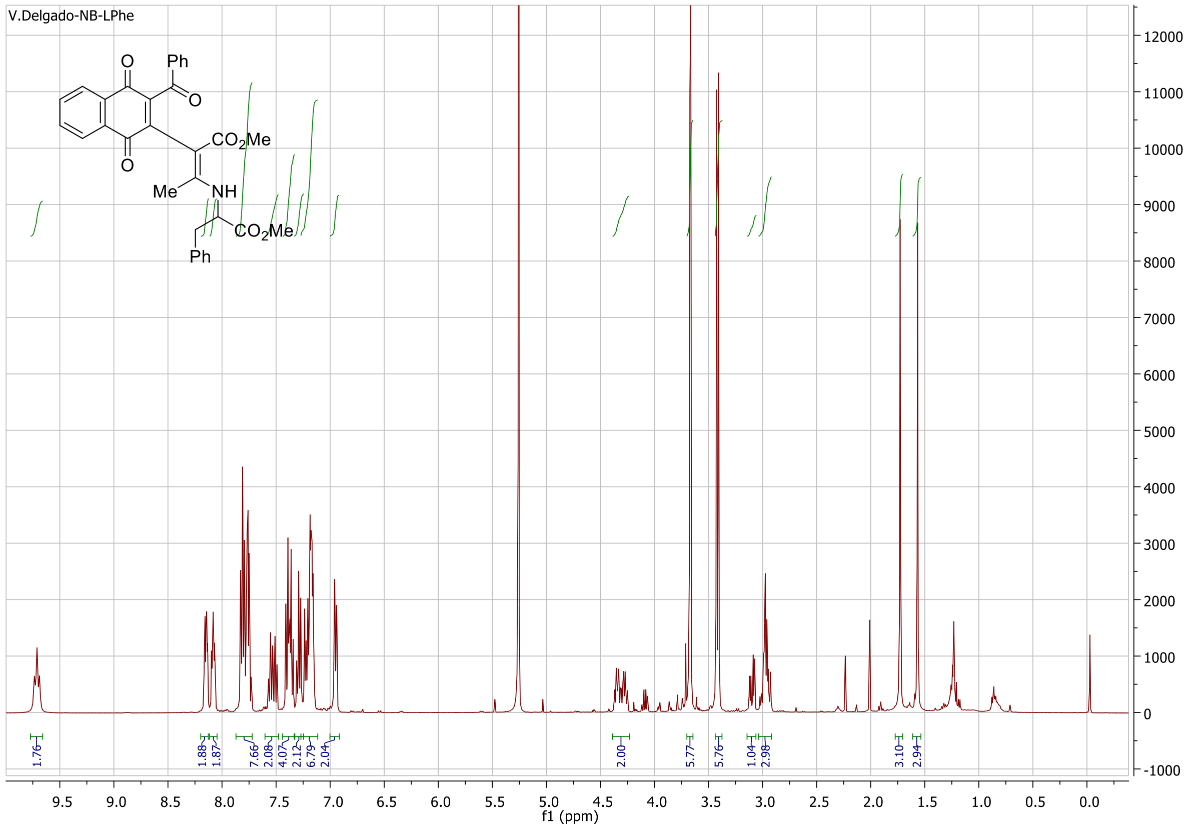
V.Delgado-NB-LPhe
O
Ph
O
CO2Me
O
Me
NH
CO2Me
Ph
1.76
1.88
1.87
7.66
2.08
4.07
2.12
6.79
2.04
2.00
5.77
5.76
1.04
2.98
3.10
2.94
f1 (ppm)

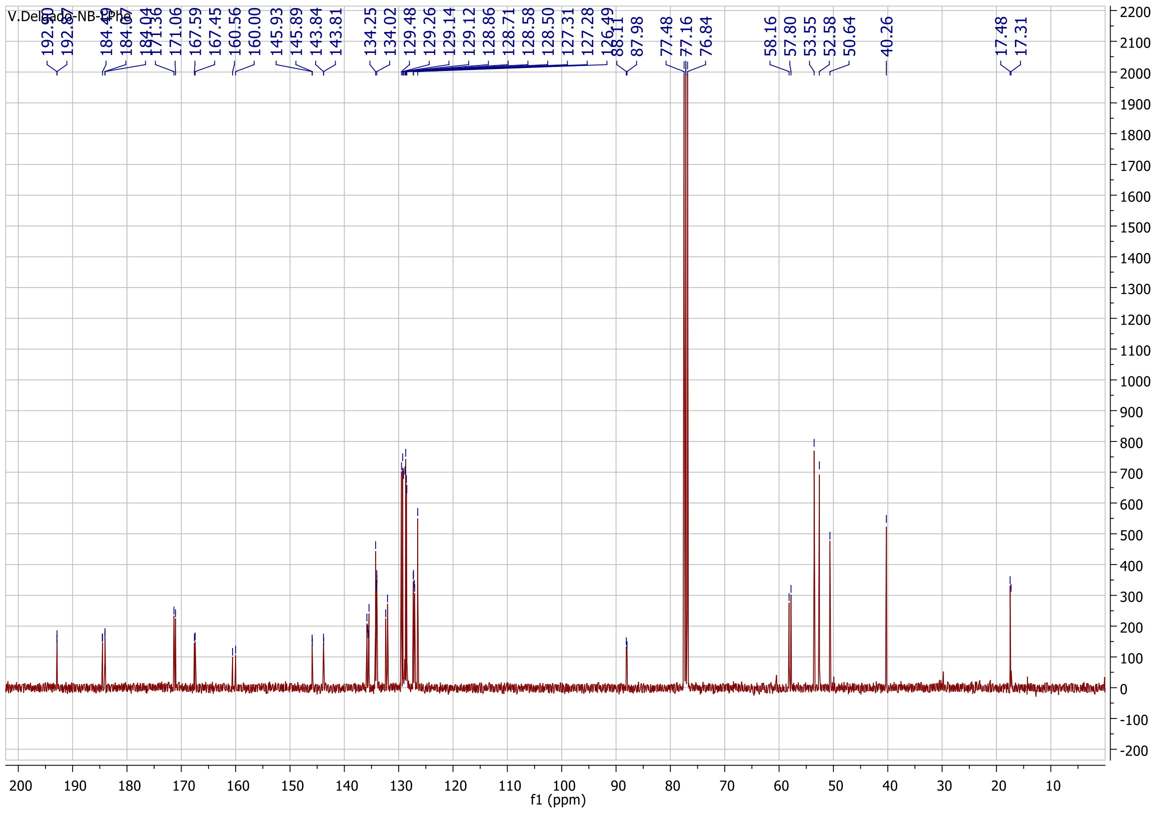
V.Delgado-NB-LPhe
192.87
184.04
171.36
171.06
167.59
167.45
160.56
160.00
145.93
145.89
143.84
143.81
134.25
134.02
129.48
129.26
129.14
129.12
128.86
128.71
128.58
128.50
127.31
127.28
126.49
88.11
87.98
77.48
77.16
76.84
58.16
57.80
53.55
52.58
50.64
40.26
17.48
17.31
f1 (ppm)

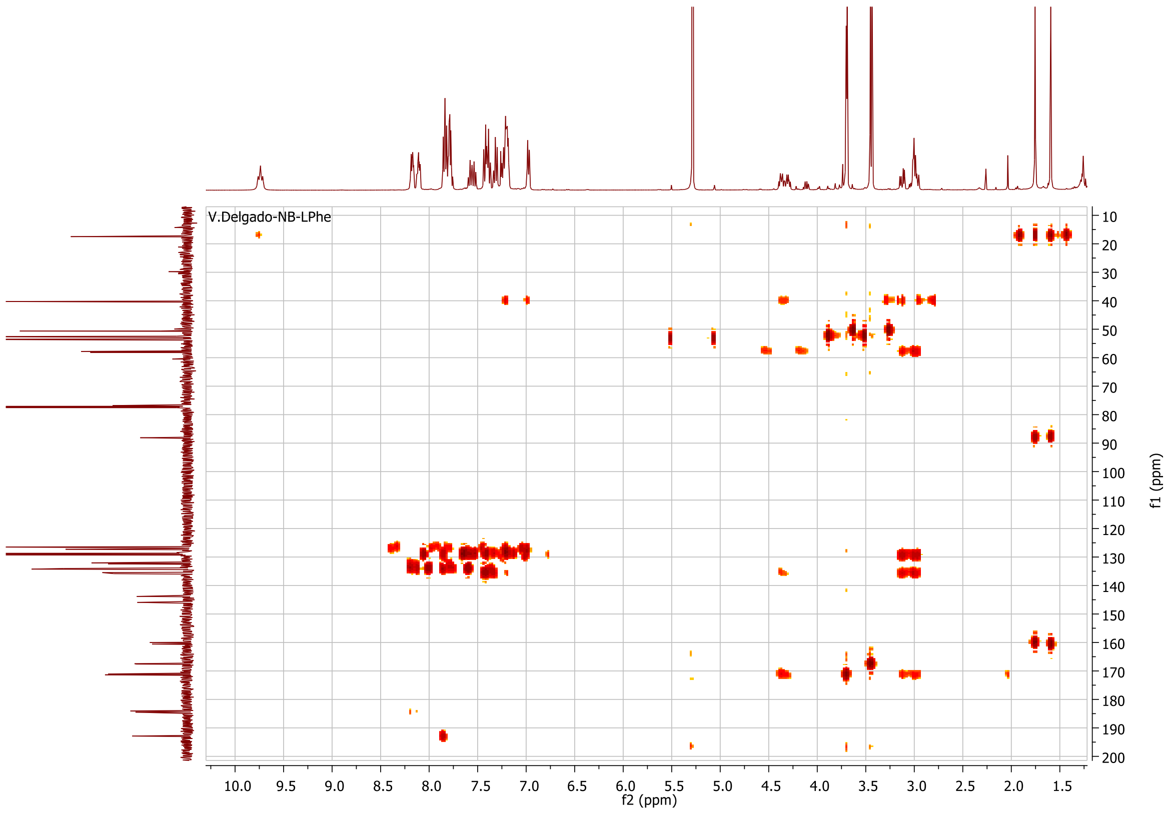
V.Delgado-NB-LPhe
f2 (ppm)
f1 (ppm)
